# Etanercept ameliorates inflammation and pain in a novel mono-arthritic multi-flare model of streptococcal cell wall induced arthritis

**DOI:** 10.1186/1471-2474-15-409

**Published:** 2014-12-04

**Authors:** Kalyan Chakravarthy, Robert Faltus, Gain Robinson, Raquel Sevilla, John Shin, Mark Zielstorff, Alan Byford, Erica Leccese, Michael J Caniga, SuChun Hseih, Shuli Zhang, Chi-Sung Chiu, Jie Zhang-Hoover, Lily Y Moy, Robbie L McLeod, Dana Stoffregen, Weisheng Zhang, Anwar Murtaza, Milenko Cicmil

**Affiliations:** Discovery Pharmacology, Merck Research Laboratories, 33 Avenue Louis Pasteur, Boston, MA 02115 USA; Imaging, Merck Research Laboratories, 33 Avenue Louis Pasteur, Boston, MA 02115 USA; Immunology Discovery, Merck Research Laboratories, 33 Avenue Louis Pasteur, Boston, MA 02115 USA; Biologics Operations, Merck Research Laboratories, 901 S. California Avenue Palo Alto, CA 94304 USA; Safety Assessment and Laboratory Animal Resources, Merck Research Laboratories, 770 Sumneytowne Pike, West Point, PA 19486 USA

**Keywords:** Rheumatoid arthritis, Animal models, Inflammation, Pain, von-Frey, SCW, TNF, Anti-TNF, Etanercept, Cytokines, Immunogenicity

## Abstract

**Background:**

The impact of anti-TNF, corticosteroid and analgesic therapy on inflammation and pain was evaluated in a novel mono-arthritic multi-flare rat Streptococcal Cell Wall (SCW) model using Etanercept, Dexamethasone and Buprenorphine.

**Methods:**

Multiple flares of arthritis were induced with an intra-articular injection of SCW in the hind ankle on day 1, followed by intravenous challenges on days 21 and 42. Inflammation and pain were monitored in the hind paws. Cytokine profiling, cell phenotyping, bioluminescence imaging and histopathological evaluation were also performed.

**Results:**

Local injection of SCW caused a rapid onset of inflammation and pain in the injected ankle which resolved within 4 days (Flare 1). Intravenous injection 20 days after sensitization resulted in an increase in ankle diameter and pain, which partially resolved in 8 days (Flare 2). The subsequent intra-venous injection in the same animals 14 days after resulted in a more chronic disease with inflammation and pain persisting over a period of 10 days (Flare 3). In Flare 2, therapeutic administration of Dexamethasone inhibited paw swelling (95%; P<0.001) and pain (55%; P<0.05). Therapeutic administration of Buprenorphine inhibited pain (80%; P<0.001) without affecting paw swelling (0%). Prophylactic administration of Etanercept in Flare 2 inhibited paw swelling (≥60%; P<0.001) and pain by ≥30%. Expression of IL-1β, IL-6, MCP-1 and CINC was reduced by >50% (P<0.001). Treatment with Etanercept in Flare 3 inhibited paw swelling by 60% (P<0.001) and pain by 25%. Prior treatment with Etanercept in Flare 2 followed by re-administration in Flare 3 led to a complete loss in the efficacy of Etanercept. Systemic exposure of Etanercept corroborated with lack of efficacy. Dexamethasone inhibited inflammation and pain in both Flares 2 and 3 (P<0.001).

**Conclusions:**

We established a novel multi-flare SCW arthritis model enabling drug intervention in different stages of disease. We show for the first time the evaluation of inflammation and pain simultaneously in this model. Etanercept and Dexamethasone inhibited inflammation, pain and proinflammatory cytokines in this model. Taken together, this model facilitates the assessment of anti-rheumatic agents targeting inflammation and pain in the multiple flare paradigm and offers a powerful tool for drug discovery.

**Electronic supplementary material:**

The online version of this article (doi:10.1186/1471-2474-15-409) contains supplementary material, which is available to authorized users.

## Background

Rheumatoid arthritis (RA) is a chronic inflammatory disease of unknown etiology that affects about 1% of the population in industrialized countries [1]. It is associated with disability, pain and significantly affects quality of life [2]. If left untreated, RA can ultimately lead to joint destruction, systemic bone loss, increased risk of fractures and other comorbidities [3, 4]. The pathogenesis of RA comprises a complex inflammatory response, involving macrophages, synoviocytes, T cells, B cells, proinflammatory cytokines and autoantibodies, causing joint damage and resulting in erosion of bone and cartilage [5, 6]. Currently, there are well established therapeutic options for treating RA, namely Non-Steroidal Anti-Inflammatory Drugs (NSAIDs) and Disease Modifying Anti-Rheumatic Drugs (DMARDs) that include biologic agents targeting cytokines, T lymphocytes and B cells [7–9]. More recently, targeting kinases with small molecule inhibitors for inflammatory disorders has been an area of intense focus for research [10]. This has led to the approval of the oral DMARD Tofacitinib, a pan-Janus kinase inhibitor, for the treatment of moderate to severe RA [11, 12].

The synovial joints of RA patients have higher levels of several proinflammatory cytokines and chemokines, the most predominant of which are: TNF, IL-1β, IL-6 and MCP-1 [13–16]. TNF is a proinflammatory cytokine that plays a central role in the pathogenesis of RA, resulting in destruction of bone and cartilage [17, 18]. Therapies targeted toward neutralizing TNF have shown substantial efficacy in RA patients and in preclinical models as well [19, 20]. Although anti-TNF therapy is a preferred strategy for the treatment of RA, some patients do not respond to anti-TNF treatment, whereas others lose the initial response over time [21]. One of the reasons for the loss of efficacy could be attributed to the generation of anti-drug antibodies (ADA) to the anti-TNF agent, that might reduce or neutralize the therapeutic effect [22].

In RA, the release of numerous proinflammatory mediators such as cytokines IL-6, IL-1β and TNF, result in an increased sensitivity to pain [23, 24]. It has also been shown that thresholds for pain and pressure are decreased in the affected joints of patients with RA [25]. The manifestation of pain is a result of both excitatory and inhibitory signals that are processed by higher brain centers [26]. Most anti-rheumatic therapeutics are effective in controlling inflammation, however further investigation is required, in order to identify novel anti-rheumatic agents that can simultaneously inhibit inflammation and pain [27]. The impact of treatment for rheumatoid arthritis is typically evaluated using American College of Rheumatology (ACR) scores, of which one of the critical components is the evaluation of pain in addition to inflammation [28]. Furthermore, Heiberg et al. [29] suggested that an improvement in pain reduction was an area of high priority and managing pain can significantly improve the quality of life for RA patients. The reduction of inflammation alone is only a partially effective strategy in the treatment of RA, as patients still present with chronic joint pain [23]. Consequently, management of pain is critical for any effective treatment paradigm for RA [27].

Animal models of Rheumatoid Arthritis have played a major role in our understanding of the mechanisms of disease pathophysiology and have supported drug discovery leading to identification of novel therapies [30–32]. Preclinical models of arthritis share many immunological, clinical and histological characteristics with human RA, however, none of them capture all the facets of the human disease [33]. Several preclinical models of arthritis such as Adjuvant Induced Arthritis (AA) and Collagen Induced Arthritis (CIA) are widely used in drug discovery [30]. These models are poly-arthritic, involving multiple joints and the disease phenotype is chronic and progressive unlike the flares and remissions observed in RA [34]. Streptococcal Cell Wall (SCW) is a rodent model of arthritis that effectively captures repeated remission and flaring phenotype, similar to RA [35]. A single intra-peritoneal injection of SCW extract PeptidoGlycan-PolySaccharide (PG-PS 10s) induces inflammation in peripheral joints with repeated phases of self-reactivating flares resembling RA [36]. However, the recurrence of reactivation is unpredictable and often difficult to control, hence this model was modified by Schwab et al. [37], in order to synchronize the flares. The modified SCW model is induced by a local intra-articular (i.a.) injection of SCW extract PG-PS 100p in the hind tarsus (flare 1) followed by a systemic intravenous (i.v.) challenge (flare 2). The model is characterized by a mono-arthritic multi-flare phenotype of two distinct remissions and flares. Inflammation is limited only to the sensitized joint with no detectable involvement of other joints, unlike other preclinical models of arthritis [38]. Demonstrating efficacy in animal models of pain is an important step in identifying novel anti-rheumatic agents that can effectively target inflammation and pain in the clinic [23]. Therefore, in addition to inflammation we have evaluated paw withdrawal threshold as a surrogate for pain. We applied the established von-Frey assay, previously described for mechanical pain assessment in preclinical animal models and in RA patients alike [25, 39]. Although, evaluation of inflammation and pain have been previously reported in other preclinical models of arthritis [40–42], to our knowledge, this is the first report investigating the clinically relevant readouts such as pain and inflammation simultaneously in the SCW model. Furthermore, we extended the model by inducing an additional flare by re-challenging the rats with an additional systemic intravenous injection of SCW (flare 3). This leads to a more persistent inflammation and mechanical pain in the previously sensitized joint. In addition, we evaluated the impact of anti-TNF, corticosteroid and analgesic therapy using etanercept, dexamethasone and buprenorphine to understand the translatability of this model in a clinical setting. The mechanisms leading to pathogenesis in the model were further delineated by histopathological evaluation, cytokine profiling, cell phenotyping and bioluminescence imaging of the arthritic joint.

Here we report distinct temporal profiles of inflammation and mechanical pain in the rat SCW model that have not been previously described. Moreover, we show that TNF could potentially be a key driver of inflammation and could in part contribute to the onset of pain in this model.

## Methods

### Animal use and care

Female Lewis rats (6–8 week old; Harlan Laboratories, Indianapolis, Indiana, USA) were acclimated for 5 days prior to the experiments and were housed under standard conditions. Female Lewis rats were used in all of these experiments due to their established susceptibility to various mediators of inflammation [43]. These experiments were conducted in accordance with federal animal care guidelines and all procedures were reviewed by the Institutional Animal Care and Use Committee (IACUC) of Merck Inc.

### Induction and assessment of the SCW model

For the induction of SCW arthritis we modified the protocol that was originally described by Schwab et al. [37]. For initial model development studies the rats were allocated to four different groups and were administered with either saline (non-arthritic; negative control), 2.5 μg, 5 μg or 10 μg of SCW extract PeptidoGlycan-PolySaccharide (PG-PS) 100p (BD Biosciences, Franklin Lakes, NJ, USA) by intra-articular (i.a.) injection into the tarsal joint to induce flare 1. On day 21, three weeks after the initial i.a. injection, rats were challenged intravenously (i.v.) with either saline (non-arthritic; negative control) or 100 μg PG-PS 100p (SCW) to induce flare 2. Based on the data from our initial studies the 5 μg dose of SCW via i.a. injection followed by 100 μg of SCW via i.v. injection was used for all subsequent experiments. In later experiments, an additional third flare (Flare 3) was induced by re-challenging the rats on day 42 with an i.v. injection of 100 μg of SCW. Inflammation and mechanical pain were assessed by measuring ankle diameter (surrogate for inflammation) using precision mechanical calipers and withdrawal threshold (surrogate for pain) using electronic von-Frey assay through the course of study.

### Withdrawal threshold: electronic von-Frey for assessing mechanical pain

Electronic von-Frey (Somedic Sales AB, Horby, Sweden) analysis was performed using methods described previously [39]. The rats were placed on an elevated grid rack under individual polycarbonate cages allowing easy access to the plantar foot surface. The tip of the von-Frey probe was brought up gently to touch the center of the paw plantar surface, and pressure was gradually increased perpendicularly at a rate of approximately 5 g/sec. The von-Frey probe records increasing pressure (grams) values, until a paw withdrawal reaction by the rat was observed. The mean of two consecutive responses per rat was recorded and used for data analysis.

### Dosing paradigm in flare 2

SCW induced arthritic rats were randomly assigned to specific treatment groups and their baseline ankle diameter and withdrawal threshold values were recorded. Test article interventions were by oral gavage or subcutaneous injection either in a Prophylactic (P) or Therapeutic (T) regimen. In the prophylactic treatment regimen, compounds were administered once daily for 10 days starting on day 20 (1 day prior to SCW intravenous challenge) ending on day 29. The therapeutic treatment regimen entailed compound administration once daily for 8 days starting on day 22 (1 day post SCW intravenous challenge) ending on day 29. Non-arthritic and SCW control rats received vehicle (PEG 400:10% Tween 80 [1:9]) orally. Etanercept (Enbrel; Amgen Inc., Thousand Oaks, CA, USA) was purchased from Myoderm Limited, Norristown, PA, USA and was reconstituted in bacteriostatic water as per manufacturer’s instructions. Etanercept (subcutaneous (s.c.); 0.25 or 1 mg/kg/day) was administered either in prophylactic or therapeutic regimens. Rats in the dexamethasone group (Sigma Aldrich, St. Louis, MO, USA) received dexamethasone (per oral (p.o.); 0.3 mg/kg/day) suspended in (PEG 400:10% Tween 80 [1:9]). Buprenorphine (Sigma Aldrich) groups received buprenorphine (p.o.; 0.05 mg/kg/day) suspended in saline in the therapeutic regimen. The dosing of compounds for all groups was stopped on day 29.

### Dosing paradigm in flares 2 and 3

In certain experiments an additional flare (Flare 3) was induced in all rats following flare 1 and flare 2. The rats were randomly assigned to two cohorts, cohort 1 and cohort 2, prior to compound administration. In cohort 1, SCW induced rats were assigned to one of the following treatment groups: etanercept (s.c.; 1 mg/kg/day), human IgG1 isotype control (s.c.; 1 mg/kg/day) or dexamethasone (p.o.; 0.3 mg/kg/day) and were dosed in the prophylactic regimen from day 21 to day 29 in flare 2. Subsequently, these rats had a drug washout period of 14 days and were treated with same doses of etanercept (s.c.; 1 mg/kg/day), human IgG1 isotype control (s.c.; 1 mg/kg/day) and dexamethasone (p.o.; 0.3 mg/kg/day) from days 41 to 51 (flare 3). In cohort 2, the rats were treated with etanercept (s.c.; 1 mg/kg/day), human IgG1 isotype control (s.c.; 1 mg/kg/day) or dexamethasone (p.o.; 0.3 mg/kg/day) in flare 3 only. Non-arthritic and SCW control rats received vehicle (bacteriostatic water) as a subcutaneous injection.

### In vivo BioLuminescence imaging (BLI)

Bioluminescence Imaging was performed using IVIS Spectrum imaging system (Perkin Elmer, Waltham, MA, USA) following methods described previously [44, 45]. Drug naïve non-arthritic controls and SCW induced rats were injected subcutaneously with a single injection of 200 mg/kg of Luminol (Sigma Aldrich) suspended in phosphate-buffered saline (PBS). Image analysis was performed using Living Image 4.0 (Perkin Elmer, Waltham, MA, USA), and average radiance (photons/second) was measured by placing a circular region of interest (ROI) centered over the SCW or non-arthritic hind tarsal joint with a second ROI placed over the contralateral tarsal joint for comparison.

### Histopathology

All the rats designated for histomorphologic assessment (post-flare 2 only) survived to scheduled study termination. At necropsy, left hindlimbs were excised distal to the hip, and fixed in 10% Neutral Buffered Formalin. The knee (with attached distal femur and proximal tibia) and hind paw (with ankle, distal tibia, tarsal bones, metatarsal and phalangeal joints) were decalcified in Immunocal (Decal Chemical Co, Suffern, NY, USA) for approximately 20 hours (knee) or 51 hours (hind paw), trimmed longitudinally (midsagittal) and placed back in decalcification solution for 2 hours (hindpaws only). After washing, the knee and hind paws from each rat were processed, sectioned, and stained with Hematoxylin & Eosin for subsequent microscopic evaluations. A veterinary pathologist scored the sections of knee and hind paws for inflammation, pannus formation, cartilage destruction, periosteal bone formation, and/or bone resorption, using severity grades: 1 = very slight, 2 = slight, 3 = moderate, 4 = marked, 5 = severe. The histomorphologic assessment was subsequently submitted for peer review by a second veterinary pathologist.

### Cytokine analysis

Ankle tissue (whole joints including synovium, bone and surrounding tissues) were excised post euthanasia and flash frozen in liquid nitrogen. The ankle tissue was pulverized and treated in a co-mixture of cell extraction solution, phosphatase and protease inhibitors. The tissue was homogenized and the supernatant was processed for cytokine expression, using Milliplex MAP 27-plex rat cytokine/chemokine magnetic bead panel (EMD Millipore, Billerica, MA, USA) on a Luminex FlexMAP 3D instrument (Luminex, Austin, TX, USA) following manufacturer instructions. Values for samples below the lower limit of the standard curve were set at the value of the lowest standard for analysis. All samples were run in duplicate or triplicate and the values are reported for technical replicates.

### Cellular phenotyping

Lymph nodes or ankle tissues were pooled for each group, prior to flow cytometry analysis. Cells from the lymph nodes were mechanically harvested to generate a single cell suspension in media. Ankle tissue was minced and digested with 0.44 U/mL Liberase enzyme and 9 U/mL dnase and made into a single cell suspension. All the antibodies (clone name in parenthesis) were purchased from BD Biosciences unless noted otherwise. The entire lymph node and ankle tissues were digested in 1800uL of the buffer, 80 uL of which was analyzed by flow cytometry to ascertain the cell population distribution in the lysate. The absolute numbers were quantified by back calculating to account for the whole lysate. Cells were blocked with anti-CD32 (D34-485) Fc block, followed by addition of either of two staining antibody cocktails: cocktail 1 containing CD45 V450 (OX-1), CD45RA FITC (OX-33), CD3 APC (1F4), CD4 pecy7 (OX-35), CD8 percp (OX-8), and cocktail 2 containing CD45 V450, CD172 PE (OX-41), anti-granulocyte FITC (HIS48), CD4 peCy7, CD163 Alexa Fluor 647 (ED2) (AbD Serotec, Kidlington, Oxford, UK). Total T cells were derived from CD45+, CD3+, CD45RA- cells from the cocktail 1 staining condition. This population was further analyzed for CD4+ and CD8+ cells. Neutrophils were derived from CD45+, anti gran hi, CD172+, CD163- cells from the cocktail 2 staining condition. T cell and neutrophil counts were analyzed relative to the starting leukocytes counts (CD45+ cells) for each assay.

### Compound exposure (PK) of etanercept

Circulating levels of etanercept in rat serum were ascertained on the Gyrolab xP instrument (Gyros AB, Uppsala, Sweden) equipped with Bioaffy 200 CD. The capture antibody used was Biotinylated-anti-TNFaRII mouse IgG2a (Clone # 22235) (R and D Systems, Minneapolis, MN, USA) and the detection antibody used was DyLight 650-conjugated anti-human IgG1 Fc Rabbit monoclonal antibody (Clone # H26-10) (Abcam, Cambridge, MA, USA). Standard curve was prepared in 50% naïve female lewis rat serum in Rexxip A (Gyros AB) with the linear range from 0.457 to 1000 ng/ml. Etanercept was quantified with Gyrolab Evaluator software.

### Data analysis

Data were analyzed and plotted using Graphpad Prism 5 (GraphPad Software Inc., La Jolla, CA, USA). Percentage inhibition for individual treatment groups are calculated by normalizing Area Under the Curve (AUC) for paw swelling and withdrawal threshold by fitting SCW vehicle groups to 100% and non-arthritic controls to 0%. AUC normalization was performed using the formula: ((Treatment – Non-Arthritic)/(SCW – Non-Arthritic)) * 100. AUC was calculated over time for flare 2 from days 21–29 and for flare 3 from days 42 to 51. Statistical significance (*P* value <0.05) was determined by two way analysis of variance for inflammation and mechanical pain analysis or by one way analysis of variance for cytokine and Bioluminescence imaging analysis followed by Bonferroni post-tests. Comparisons were made for all drug treated groups versus non-arthritic treated with appropriate vehicle (represented by *) or SCW treated with appropriate vehicle (represented by ^) groups. All values are expressed as mean ± SEM, unless otherwise noted. Relative change of ankle diameter and withdrawal threshold in SCW injected rats as compared to non-arthritic controls is represented by Δ.

## Results

### Establishment of the SCW mono-arthritic multi-flare model

**Figure 1:**
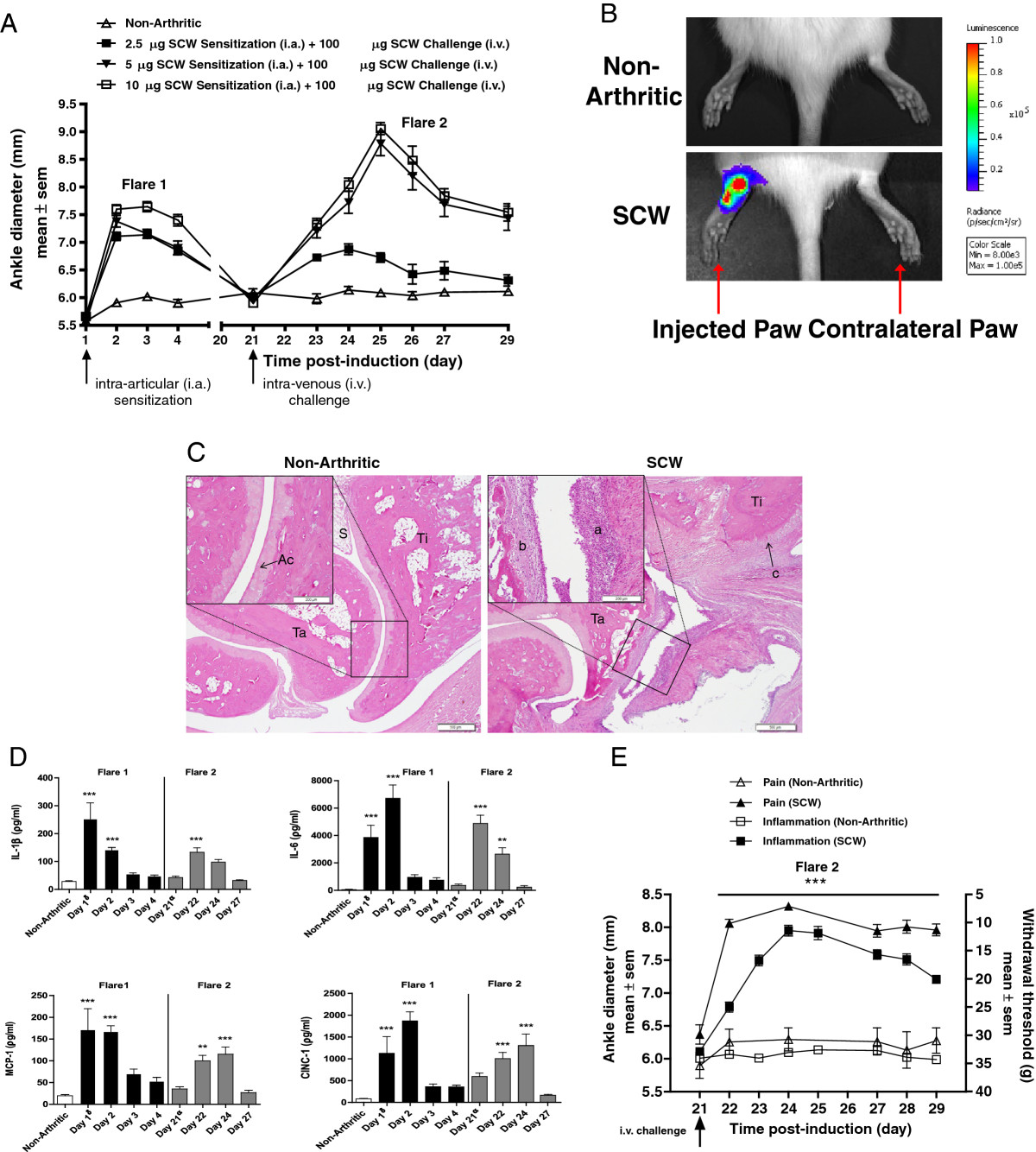
**Establishment of the SCW mono** ***-*** **arthritic multi** ***-*** **flare model. (A)** Inflammation of the ankle joint followed over a 29 day period after administration of increasing doses (2.5 μg, 5 μg, or 10 μg) of SCW sensitization via i.a. delivery of antigen (day 1), followed by a systemic challenge (fixed dose 100 μg) on day 21. Values are mean ± SEM of 8-11 rats per group. **(B)** Bioluminescence imaging comparing non-arthritic and SCW rat ankle joint 6 hours following sensitization with 5 μg SCW. **(C)** Representative histomorphological sections of rat ankle joints collected on day 29 following SCW sensitization comparing Non-Arthritic and SCW rat paw. Points of interest are Tarsus (Ta), Tibia (Ti), slight inflammation (a), slight pannus (b), slight periosteal ossification (c), Synovium (S) and Articular cartilage (Ac). **(D)** Kinetics of cytokine expression in local injected ankle joint. Protein exudates were extracted from non-arthritic and arthritic ankle joints at various time points following i.a. sensitization of 5 μg of PGPS (Flare 1), and following systemic challenge of 100 μg PGPS (Flare 2). Levels of Interleukin 1 beta (IL-1β), Interleukin 6 (IL-6), Monocyte Chemotactic Protein-1 (MCP-1) and Cytokine-Induced Neutrophil Chemoattractant (CINC) were evaluated (shown from top left to lower right respectively). Values are mean ± SEM of 7-16 rats per group. δ Day 1 time point was at 6 hours post i.a. sensitization; α Day 21 time point was at 6 hours post i.v. challenge **(E)** Simultaneous evaluation of pain and inflammation. A composite of 4 independent studies showing inflammation (ankle diameter; Y axis) and pain (withdrawal threshold; Z axis) over time (days; X axis) following systemic i.v. challenge in Flare 2. Values are mean ± SEM of 6-8 rats per group. ** = P < 0.01 versus Non-Arthritic; *** = P < 0.001 versus Non-Arthritic.

Increasing doses (2.5 μg, 5 μg and 10 μg) of SCW were administered via i.a. injection into the hind tarsal joint on day 1 (Figure 1A). The local injection resulted in a marked increase in ankle diameter that peaked on day 2 (24 hr post i.a. injection), followed by a continued decline in ankle diameter by day 4 in all 3 dose groups (flare 1). To confirm delivery of the antigen to local joint space, hind limbs of the rats injected with 5 μg SCW, were assessed by BLI imaging for myeloperoxidase activity using luminol 6 hr post SCW sensitization. Negative controls included contralateral paws or hind limbs from non-arthritic controls. Significantly higher bioluminescence at the site of SCW injection (1.1 * 10^6^ ± 1.7 * 10^5^ photons/second; *P* < 0.001) was detected compared to the contralateral and non-arthritic control ankles, confirming local delivery of SCW to the ankle joint (Figure 1B). The rats from the SCW injected groups were challenged on day 21 via a systemic i.v. injection of 100 μg SCW. A dose dependent increase in ankle diameter was observed in the 2.5 μg, 5 μg and 10 μg SCW treated groups (Δ0.65, Δ2.7, Δ3 mm, respectively) compared to the non-injected controls with a maximal response between days 24–25. This was followed by a gradual decrease in hind paw swelling over a period of 4 days (flare 2). The dose combination of 5 μg SCW at sensitization and 100 μg SCW at challenge resulted in robust and reproducible ankle edema in both flares 1 and 2 compared to contralateral and non-arthritic control ankles. This dose combination was used for all further experiments.

### Cellular phenotyping in ankle tissue and lymph nodes

Based on the previously established role of neutrophils and T cells in the pathogenesis of the model [46], we phenotyped these cells in the local ankle joint and lymph nodes by flow cytometry. We observed an increase in neutrophils and T cells in the injected ankle joint 24 hr post induction of flare 1 compared to non-arthritic controls. The infiltrating T cells in the local joint were primarily CD4^+^, however, CD8^+^ T cells were not observed at this time-point. In flare 2 at 24 hr post induction, a lower number of neutrophils were observed in the local joint compared to flare 1. Interestingly, at this time point we detected a two fold increase in the number of T cells in the popliteal lymph node, proximal to the site of sensitization compared to non-arthritic controls (Table 1).

**Table 1:** **Cellular phenotyping of neutrophils and T cells in local and systemic compartments^**

Tissue	Cell type	Non-arthritic	Flare 1 (24 hr post i.a. injection)	Flare 2 (24 hr post i.v. injection)
Ankle joint	Neutrophils	3561	6.4* 10^5^	5.6* 10^4^
	CD3+ T cells	1413	1.6* 10^5^	2.6* 10^4^
Lymph node	Neutrophils	BLQ	BLQ	BLQ
	CD3+ T cells	9.1* 10^6^	10.1* 10^6^	19.9* 10^6^

^The total number of cells in the injected ankle joint and lymph node compartments as ascertained by flow cytometry using methods described above. Values calculated from pooled tissue samples (n = 4 animals/group). BLQ: Below the Level of Quantification.

### Histopathological evaluation

Histomorphologic assessment was limited to the hind limbs post flare 2 rats only euthanized on day 29 (Figure 1C). SCW-injected tarsal joints had significant injury, localized primarily to the tibio-tarsal and proximal intertarsal joints and associated tibial and tarsal bones, characterized by slight inflammation and slight to moderate pannus formation (associated with overt bone loss). A few rats in this group also had slight focal periosteal bone formation. The remaining hindlimb joints (stifle proximal to the injected paw, and contralateral hind paw and stifle) had no histomorphologic findings. Hind paws from the non-arthritic control rats served as negative controls, and there were no histomorphologic findings.

### Kinetics of cytokine expression in local sensitized ankle joint

Next, the kinetics of cytokine and chemokine expression were examined to delineate inflammatory pathways involved in the flaring mechanism in the model. Arthritis was induced as described above and the injected and contralateral ankles were excised post euthanasia at the following time points: in flare 1 on days 1 (6 hr post i.a. sensitization), 2, 3, 4 and in flare 2 on days 21 (6 hr post i.v. challenge), 22, 24 and 27. Using the methods described above, ankle joint homogenates were analyzed for cytokine expression. We observed a significant up regulation of the cytokines IL-6, IL1-β, MCP-1 and CINC-1 in the local injected ankle joint in both flares 1 and 2 with diverse temporal profiles. In flare 1, IL-1β peaked (253 ± 58 pg/ml; *P* < 0.001) on day 1 (6 hr post i.a. sensitization) and continued to decline over the period of 4 days. IL-6 increased on day 1 (6 hr post i.a. sensitization) and reached a peak (6720 ± 961 pg/ml; *P* < 0.001) on day 2. MCP-1 peaked (171 ± 49 pg/ml; *P* < 0.001) on day 1 and CINC-1 peaked (1870 ± 205 pg/ml; *P* < 0.001) on day 2 and their levels started to decline by days 3 and 4. In flare 2, statistically significant up regulation was observed in IL-1β (133 ± 16 pg/ml; *P* < 0.001) and IL-6 (4886 ± 599 pg/ml; *P* < 0.001) on day 22, with a trend of decline on days 24 and 27. Both MCP-1 (115 ± 16 pg/ml; *P* < 0.001) and CINC-1 (1304 ± 263 pg/ml; *P* < 0.001) expression peaked on day 24 and declined to baseline by day 27 (Figure 1D).

### Simultaneous evaluation of pain (withdrawal threshold) and inflammation (ankle diameter)

Using the von-Frey test for pain measurement, we sought to align the model with clinical symptoms. We studied the mechanical pain response elicited by the rats in flare 2. Figure 1E illustrates the composite scores of inflammation and pain from 4 independent studies. Pain response assessed by withdrawal threshold (Z axis) is plotted versus the inflammation measured by ankle diameter (Y axis) for the same rats over days 21–29 following i.v. systemic challenge of SCW (X axis). A robust and statistically significant pain response was observed in SCW induced rats reaching a peak between day 22–24 and persisting through day 29 (*P* < 0.001), compared to non-arthritic control rats. Furthermore, inflammation as assessed by paw swelling, developed gradually, reaching a peak on day 24 (*P* < 0.001) and showed a trend toward decline on day 29. Taken together, the data suggests that pain may precede inflammation and continue to persist up to day 29, whereas inflammation starts to decline by day 29. The robust inflammation (Δ ~2.0 mm) and pain (Δ ~20 g) window between non-arthritic and SCW groups enabled further pharmacological evaluation of compounds of interest. The inflammation and pain response in the contralateral paws of the SCW rats did not change from their baseline values and the data was similar to non-arthritic controls (data not shown).

### Effect of corticosteroid and analgesic therapy on inflammation and pain (flare 2)

**Figure 2:**
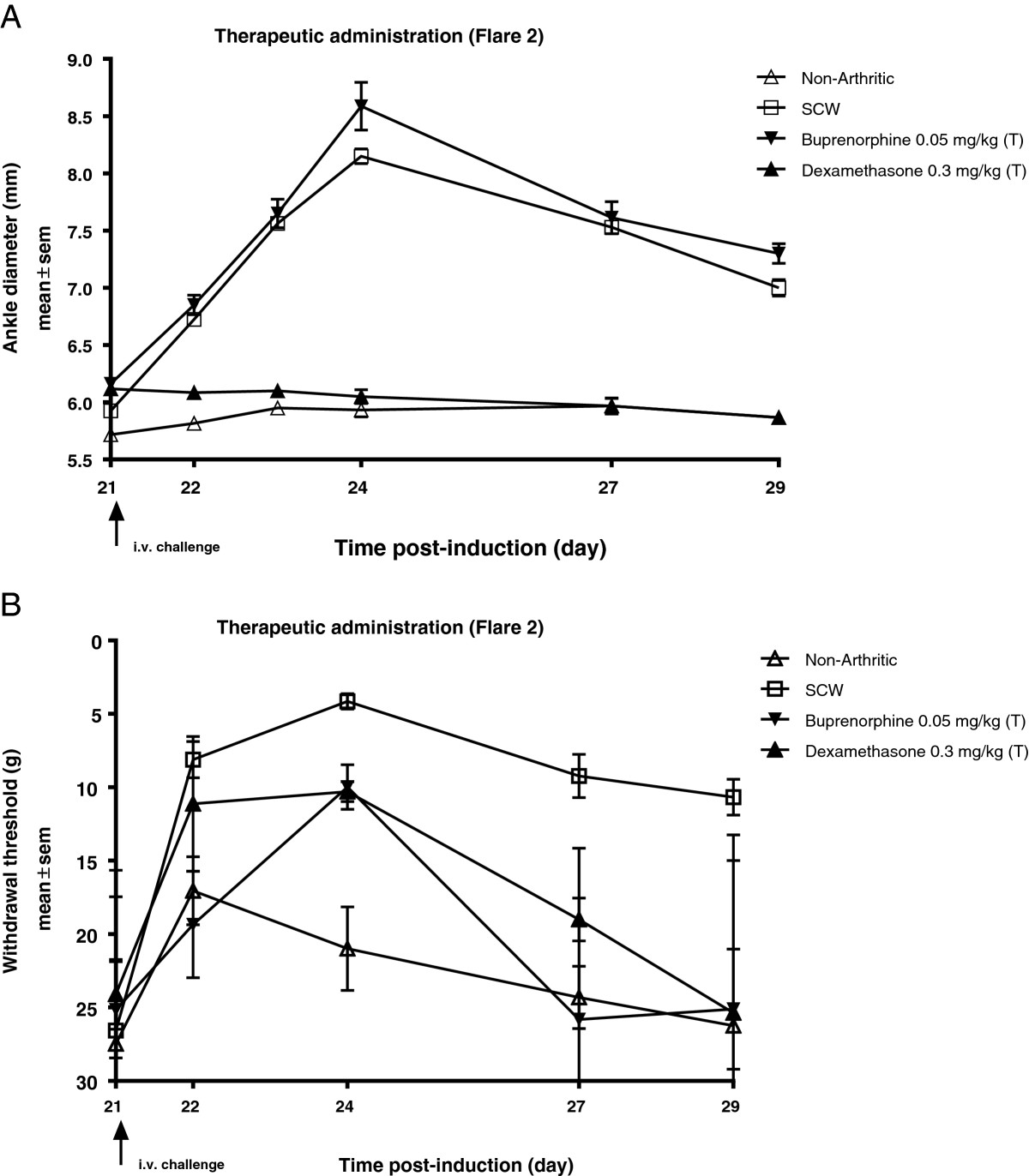
**Effect of corticosteroid and analgesic therapy on inflammation and pain (flare 2). (A)** Ankle diameter post systemic i.v. challenge followed until day 29 and **(B)** Paw withdrawal threshold values plotted for each group. Values are mean ± SEM of 8–11 rats per group. (T) therapeutic.

To further understand the pathogenic mechanisms amenable to drug treatment, we evaluated the effects of dexamethasone and buprenorphine on inflammation and mechanical pain. Both dexamethasone (0.3 mg/kg/day) and buprenorphine (0.05 mg/kg/day) were administered once daily in the therapeutic regimen. Dexamethasone administration resulted in the significant inhibition of paw swelling by 95% (*P* < 0.001) and pain by 55% (*P* < 0.05) compared to the SCW vehicle treated rats. On the other hand, buprenorphine administration significantly inhibited pain by 80% (*P* < 0.001) and did not have any effect on paw swelling (0%) (Figure 2A-B).

### Etanercept inhibits inflammation and pain (flare 2)

**Figure 3:**
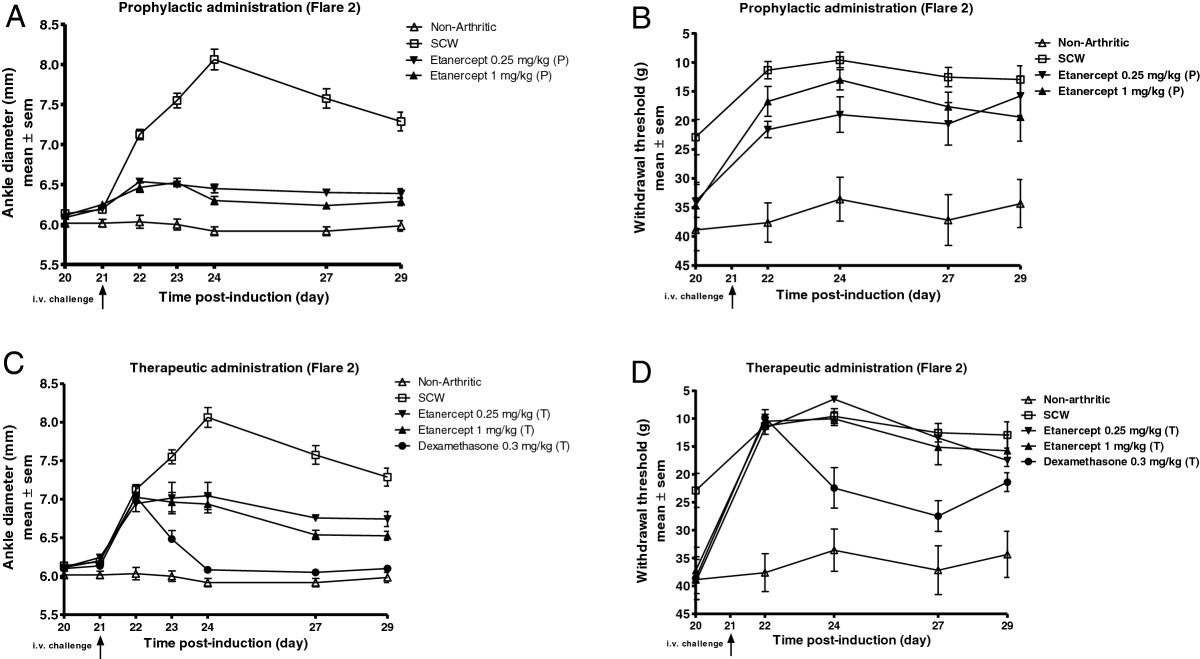
**Etanercept inhibits inflammation and pain in flare 2. (A)** Effect on inflammation of etanercept administration at 0.25 mg/kg and 1 mg/kg when dosed prophylactically; **(B)** effect on mechanical pain. **(C)** Effect on inflammation of etanercept administration at 0.25 mg/kg and 1 mg/kg when dosed therapeutically; **(D)** effect on mechanical pain. Effect on inflammation of dexamethasone dosed therapeutically **(C)**; effect on pain **(D)**. Values are mean ± SEM of 6–8 rats per group. (P) prophylactic; (T) therapeutic.

To further investigate the role of TNF in the model, the effect of etanercept was evaluated in flare 2. Etanercept (0.25 mg/kg/day or 1 mg/kg/day) was administered in both prophylactic (day 20, 24 hr prior to i.v. challenge) and therapeutic (day 22, 24 hr after i.v. challenge) regimens. In the prophylactic regimen, etanercept at 0.25 mg/kg significantly inhibited inflammation by 70% (*P* < 0.001) and had a modest effect on mechanical pain (37%; *P* < 0.05). The 1 mg/kg dose of etanercept significantly inhibited inflammation by 76% (*P* < 0.001) and reduced pain by 24%, compared to SCW vehicle treated rats (Figure 3A-B; Table 2). Conversely, in the therapeutic regimen, etanercept at 0.25 mg/kg and 1 mg/kg significantly inhibited inflammation by 44% (*P* < 0.001) and 52% (P < 0.001) respectively, however both the doses were ineffective in suppressing pain. Therapeutic administration of positive control dexamethasone (0.3 mg/kg/day) inhibited inflammation by 84% (*P* < 0.001) and pain by 39% (*P* < 0.05) (Figure 3C-D; Table 2).

**Table 2:** **Percentage inhibition in area under the curve values normalized to non-arthritic and SCW vehicle groups for inflammation and mechanical pain in flare 2 for the experiment illustrated in Figure** 3 *

	Inflammation (ankle diameter)		Pain (withdrawal threshold)	
	Prophylactic	Therapeutic	Prophylactic	Therapeutic
Etanercept 0.25 mg/kg	70% (*P* < 0.001)	44% (*P* < 0.001)	37% (*P* < 0.05)	0% (ns)
Etanercept 1.0 mg/kg	76% (*P* < 0.001)	52% (*P* < 0.001)	24% (ns)	0% (ns)
Dexamethasone	nd	84% (*P* < 0.001)	nd	39% (*P* < 0.05)

*The values of statistical significance against SCW vehicle groups are represented in parenthesis. In all cases, non-arthritic controls were significantly different from SCW vehicle groups (*P* < 0.001). Values are calculated from 8–11 rats per group. ns: not significant; nd: not determined.

### Etanercept inhibits production of cytokines IL-6, IL-1β, MCP-1 and CINC-1 (flare 2)

**Figure 4:**
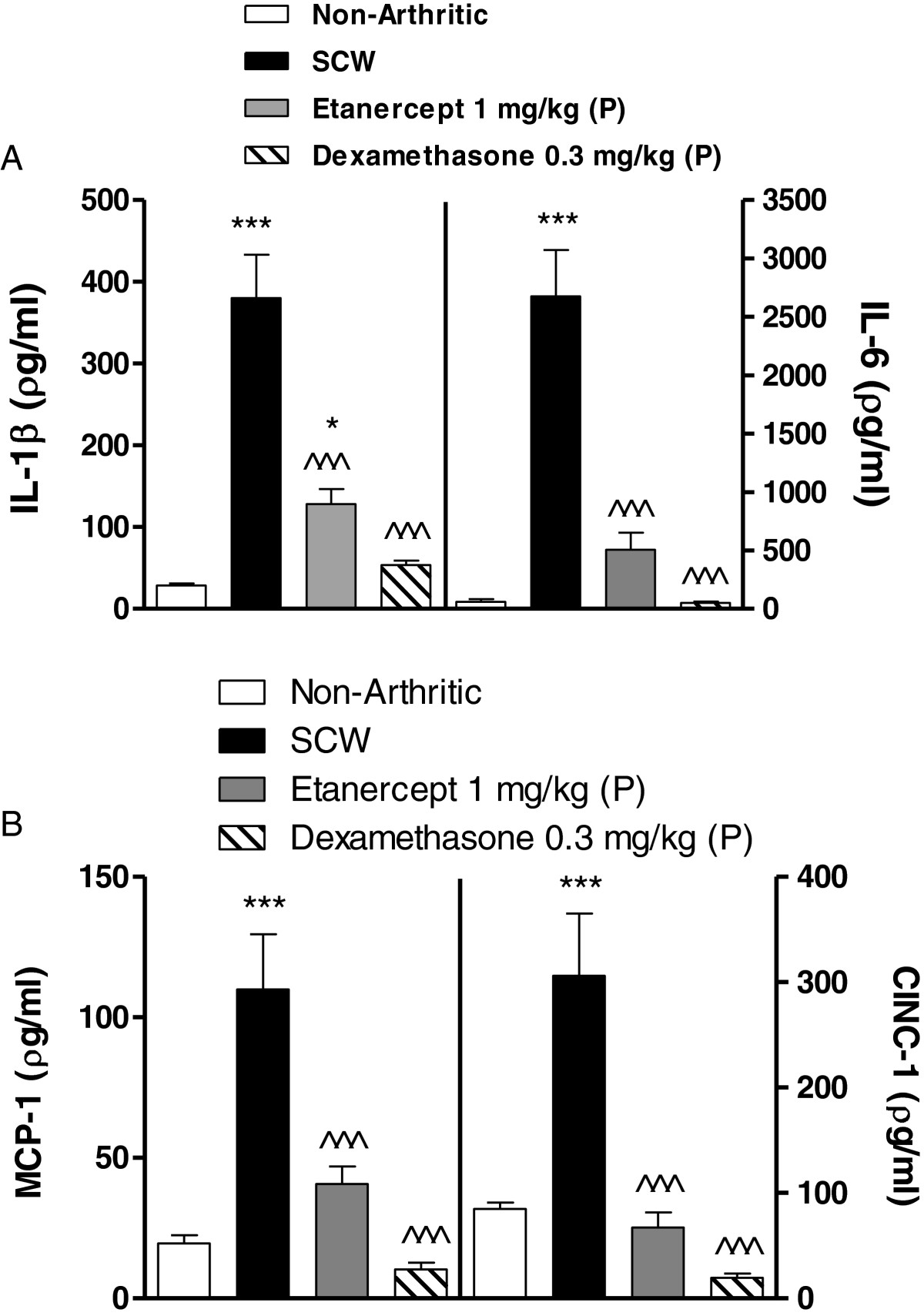
**Etanercept inhibits production of cytokines IL-6, IL-1β, MCP-1 and CINC-1 in the arthritic paw (flare 2). (A)** Effect of etanercept (1 mg/kg prophylactically) on levels of Interleukin-1 beta (IL-1β), Interleukin-6 (IL-6) and **(B)** Effect of etanercept (1 mg/kg prophylactically) on levels of Monocyte Chemotactic Protein-1 (MCP-1) and Cytokine-Induced Neutrophil Chemoattractant-1 (CINC-1) from ankle joint homogenates collected on day 22, 24 hours post induction of the flare 2. Values are mean ± SEM of 12–16 rats per group. * = *P* < 0.05 versus Non-Arthritic; *** = *P* < 0.001 versus Non-Arthritic; ^^^ = *P*<0.001 versus SCW. (P) prophylactic; (T) therapeutic.

As illustrated in Figure 4A-B, we ascertained the effect of etanercept on the production of cytokines previously shown to be up-regulated in the model. A group of SCW induced rats were administered a subcutaneous dose of etanercept (1 mg/kg/day) prophylactically and were sacrificed on day 22, one day after the induction of second flare. In the local ankle joint, we observed a statistically significant reduction in cytokine expression levels of IL-1β (72%), IL-6 (83%), MCP-1 (77%) and CINC-1 (100%) with etanercept administration (*P* < 0.001). Positive control dexamethasone (0.3 mg/kg/day) resulted in near maximal suppression of the same cytokines (*P* < 0.001).

### Induction of an additional flare (flare 3): evaluation of inflammation and pain

**Figure 5:**
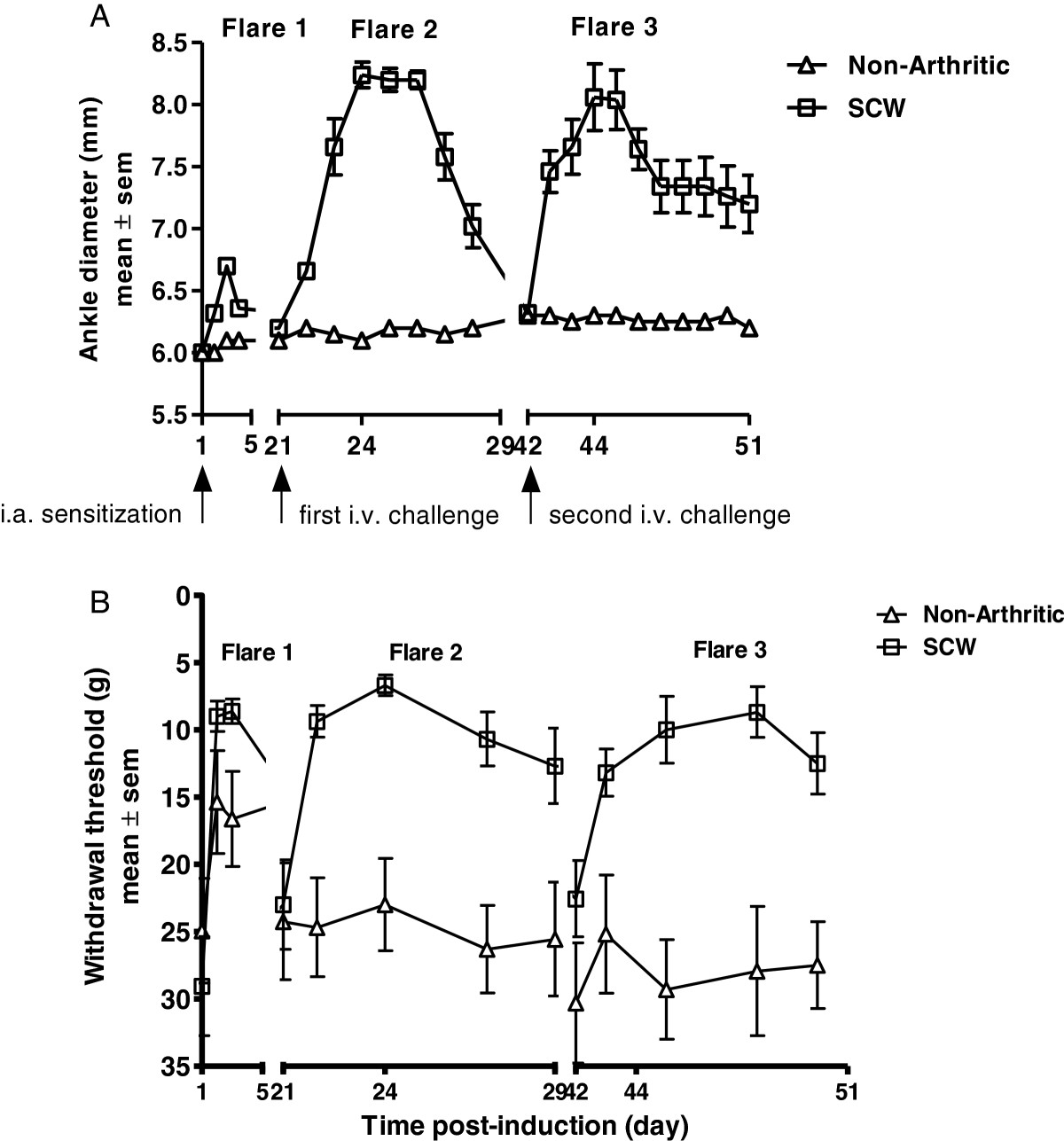
**Induction of an additional flare (flare 3): evaluation of inflammation and pain. (A)** Inflammation of the ankle joint following intra-articular sensitization (day 1; flare 1), the first intravenous challenge (day 21; flare 2) and the second intravenous challenge (day 42; flare 3). **(B)** Mechanical pain response (withdrawal threshold) in the same rats over the same time period. Values are mean ± SEM of n =8 rats per group.

We attempted to capture the chronic phase of the disease by extending the model to a third flare, by re-challenging the SCW sensitized rats with a second i.v challenge of SCW (flare 3). We observed that the inflammation in flare 3 (Δ 2 mm; *P* < 0.001) peaked on day 44 (72 hr post re-challenge), similar to inflammation kinetics observed in flare 2. However, inflammation in flare 3 continued to persist for 10 days until study termination on day 51 (Figure 5A). Figure 5B illustrates the mechanical pain response in the sensitized paw. Similar to the kinetics of inflammation, the pain response following reactivation continued to increase significantly and remained elevated throughout the 10 day period, with a maximal response observed on day 45 (96 hr post re-challenge) (*P* < 0.01). As anticipated non-arthritic control rats showed an increase in the pain response post local i.a. sensitization with saline. However, systemic i.v. injections had no effect on the pain in the non-arthritic controls and the response was similar to their baseline values. The cytokine expression profiles of IL-1β, IL-6, MCP-1 and CINC-1, were similar to those observed in flare 2 (data not shown).

### Comparative evaluation of repeated administration of etanercept in both flares 2 and 3 versus administration in flare 3 alone

**Figure 6:**
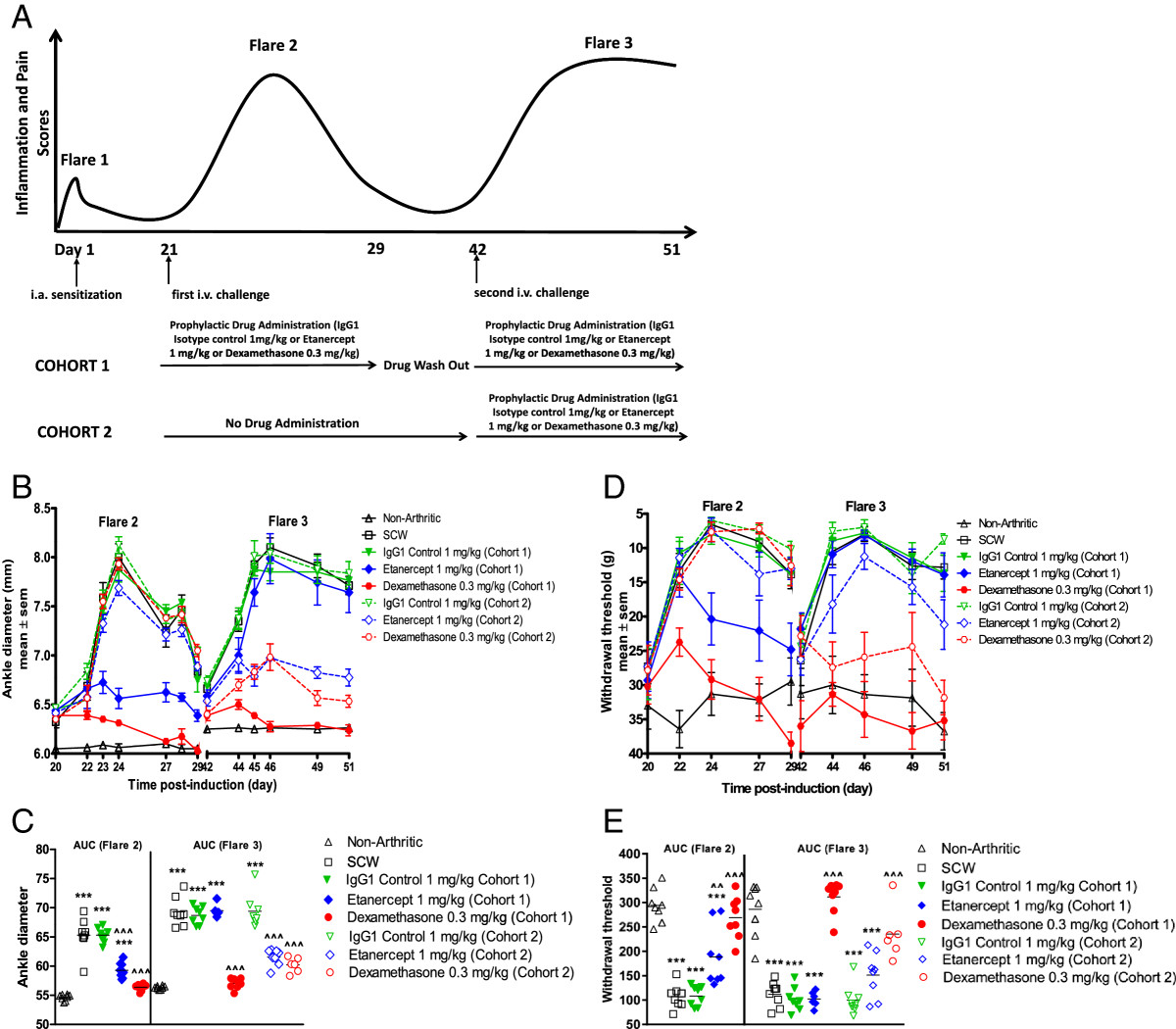
**Effect of Etanercept in flares 2 and 3 versus administration in flare 3 alone. (A)** A schematic representation of the dosing paradigm for groups tested in the multi-flare model of SCW. Inflammation and pain values were recorded in all groups throughout the study. Two separate cohorts underwent different dosing paradigms to investigate the response to single or repeated drug administration in each flare. **(B)** Effect on inflammation of prophylactic administration of etanercept in cohorts 1 and 2; **(C)** Corresponding AUC calculations for inflammation. **(D)** mechanical pain responses measured concurrently with inflammation in the same rats in cohorts 1 and 2. **(E)** Corresponding AUC calculations for the pain response. Effect of prophylactic administration of dexamethasone on inflammation and pain in both cohorts (6B-E). Values are mean ± SEM of n= 8 rats per group. *** = *P*<0.001 versus Non-Arthritic; ^^ = *P*<0.01 versus SCW; ^^^ = *P*<0.001 versus SCW.

Next, we compared the efficacy of etanercept (1 mg/kg/day) in flare 2 versus flare 3, to delineate differences in pathogenic mechanisms between the two flares. In addition, we wanted to investigate whether prior treatment with etanercept in flare 2 would have a sustained or improved efficacy when re-administered in flare 3 with an intervening drug washout period.As depicted in Figure 6A, Cohort 1 received etanercept in flare 2 followed by a 14 day drug washout period and was re-administered the same dose of etanercept in flare 3. Conversely, Cohort 2 received etanercept in flare 3 only. In both the cohorts etanercept was administered prophylactically one day prior to the induction of flare 2 or flare 3.

In Cohort 1 rats (flare 2) treatment with etanercept suppressed inflammation by 56% (*P* < 0.001) and inhibited mechanical pain by 44% (*P* < 0.01), as previously observed. The rats treated with etanercept only in flare 3 (Cohort 2) showed 60% (*P* < 0.001) inhibition of inflammation and a modest effect on pain (23%). Our data suggests that when dosed in individual flares, the efficacy of etanercept on inflammation and pain in flare 2 and 3 was comparable (Figure 6B-E).

We also investigated inflammation and pain in Cohort 1 rats that received etanercept treatments in both flare 2 and 3 with an intervening drug washout period of 14 days. Interestingly, we observed a complete loss of etanercept efficacy on both inflammation and pain upon re-administration in flare 3 (Figure 6B-E). The loss of efficacy upon re-administration of etanercept could be due to potential immunogenicity as a result of antibody production against etanercept, leading to its enhanced clearance and consequently reduced systemic exposure. To test this hypothesis we measured the circulating levels (PK) of etanercept in serum at the termination of the study on day 51. Circulating levels of etanercept were below the levels of detection in these animals. In contrast, pharmacologically efficacious levels of circulating etanercept (2.4 ± 1.5 ng/ml) were detected in rats from Cohort 2 that had been treated in flare 3 only. Positive control dexamethasone (0.3 mg/kg/day) significantly inhibited inflammation and pain in both Cohorts 1 and 2 (*P* < 0.001). Treatment with human IgG1 isotype control in both the cohorts had no effect on inflammation or pain and the data was similar to vehicle treated SCW rats.

## Discussion

RA is characterized by a complex interplay of various pathogenic mechanisms leading to inflammation and pain [47]. Animal models of arthritis can be effective tools to investigate these mechanisms and delineate pathways that might help predict the successful outcome of novel therapeutics in the clinic. The previously described mono-arthritic SCW model in rats captures the relapsing and remitting flares of the disease, similar to RA [35]. Typically, the model is characterized by two flares that resolve over time [37, 48]. Intra-articular injection of SCW antigen to local joint results in mono-arthritis with inflammation limited only to the sensitized joint, even after systemic challenge with the antigen. The first flare induced by an intra-articular injection of SCW results in mild paw swelling that peaks 24 hrs post sensitization (flare 1) and resolves over 72 hours. The second flare induced by an intravenous challenge with SCW (typically three weeks later) results in a pronounced onset of paw swelling reaching peak on day 3 after i.v. challenge (flare 2) and resolves over a 5 day period. Previous studies by Schimmer et al. [46], showed that the early phase of the model can be triggered by Th2 cells and neutrophils. They have also demonstrated that neutrophils were involved in addition to T cells in the reactivation of flares. In addition, the model is dependent on multiple proinflammatory cytokines including TNF and IL-1, as assessed by specific anti-cytokine therapy and gene expression analysis [49, 50]. In order to capture the chronic disease phenotype, we have developed an extended version of the model with the induction of an additional flare (flare 3). The third flare appears to exhibit a chronic disease phenotype not previously described. The mono-arthritic SCW model is a robust model with reported incidence of arthritis in 90-100% of the rats [34]. In line with literature, we were successful in inducing arthritis in 100% of the animals in all the three flares.

Histological assessment demonstrated that inflammation, pannus formation, degeneration of cartilage, periosteal bone formation, and/or bone resorption in SCW rats was limited to the sensitized hind paw, there were no histomorphologic findings in the proximal hind limb joint tissues and the contralateral hind limb and hind paw. We also observed that the gross histological damage in the model was mild to moderate compared to CIA or AA models and this is in agreement with current literature [34, 48]. An additional attractive feature of this model, is that the disease severity and progression is less severe when compared to other rodent models of arthritis [34, 38]. We observed that even in the extended flare 3, the disease was mild and the rats maintained a healthy status throughout the course of the study.

Neutrophils have been shown to play a major role in both flare 1 and 2. Depleting neutrophils by an anti-neutrophil antibody has been shown to be efficacious in the model [49]. Our results show the presence of neutrophils in flare 1 as determined by flow cytometry and in flare 2 as assessed by histology. In addition to neutrophils, T cells also have been shown to contribute to the pathogenesis of disease [38]. 24 hr post induction of both flare 1 and 2, we observed an up regulation of T cells in the ankle joint. The infiltrating T cells in the local joint belonged to the CD4^+^ subpopulation, however CD8^+^ T cells were not detected at this time-point. These findings were also corroborated by histological analysis of samples collected on day 29. A two fold increase in T cells was observed in the draining lymph node, 24 hr post induction of flare 2 compared to flare 1 and non-arthritic controls (Table 1). We further characterized the production of multiple proinflammatory cytokines and showed increased levels of IL-1β, IL-6, MCP-1, CINC-1 in the local joint. The role of TNF, IL-1β, IL-6, MCP-1 and CINC-1 in the pathogenesis of SCW has been demonstrated previously [50, 51]. Moreover, neutralizing antibodies to TNF, IL-1α/β and MCP-1 have been shown to inhibit inflammation, thereby further confirming the role of these cytokines in the model [46, 49]. Although, we expected to detect TNF in the arthritic joint homogenates, this cytokine could not be quantified perhaps due to its transient kinetics or the sensitivity of the assay. The presence of IL-1β and IL-6 in the arthritic joint of SCW rats and the role of these proinflammatory cytokines in inducing pain in addition to inflammation in RA, led us to investigate the kinetics of pain in our model [15, 24, 51].

In RA, pain experienced by patients involves the complex interplay of nociceptive and inflammatory processes [23]. Changes in joint pathology often result in an increased sensitization of primary sensory neurons and central sensitization, due to the changes in ascending or descending modulatory pathways [52]. Physical changes in the rheumatic joint (tissue edema, biochemical changes, inflammatory mediators, nociceptor activation) all can cause a decrease in the threshold for pain [53]. Furthermore, pain is an important clinical feature of RA and its assessment is incorporated in the ACR scoring paradigm [28]. The mono-arthritic SCW model allows us to concurrently assess pain and inflammation following drug intervention, facilitating the discovery of novel anti-rheumatic agents. Simultaneous assessment of inflammation and pain has been previously described in the polyarthritic mouse CIA model [54]. However, the distinctive mono-arthritic phenotype of the SCW model offers an advantage over the poly-arthritic models, as we were able to monitor mechanical pain and inflammation in the sensitized joint, with the contralateral paw serving as an internal negative control.

To further characterize our model, we profiled a panel of therapeutic agents such as, etanercept, dexamethasone and buprenorphine. We selected these agents based on their established efficacy in other preclinical models of arthritis [55–57]. The specific doses used for each drug in these studies were selected based on multiple dose response studies conducted internally (data not shown). In flare 2, treatment with anti-TNF agent, etanercept resulted in a significant inhibition of paw swelling in both prophylactic and therapeutic regimens. Interestingly, prophylactic treatment with etanercept showed a modest decrease in mechanical pain, which was statistically significant. However, therapeutic treatment of etanercept had no effect on pain. Our data suggests that TNF may play a bigger role in the onset and progression of paw swelling, whereas other cytokines such as IL-1β, IL-6, or other mechanisms in addition to TNF, could also contribute to pain. Currently, efforts are underway to further understand the roles of these mechanisms in the multi-flare model pathophysiology. Therapeutic treatment with corticosteroid dexamethasone was more effective in inhibiting paw swelling and mechanical pain compared to etanercept. Prophylactic administration of analgesic buprenorphine significantly inhibited pain but had no effect on inflammation. Taken together, these results suggest that inflammation and pain can be distinguished and evaluated separately in the SCW model. Histomorphological evaluation of the local joints corroborated with the efficacy data, showing reduced severity of inflammation and pannus formation after treatment with etanercept and dexamethasone (data not shown). Our internal data also show similar efficacy profiles of etanercept and dexamethasone in the rat CIA model on inflammation (data not shown).

We further extended the model to capture the chronic phase of the disease (RA), by inducing an additional flare via a second systemic antigen challenge of SCW, characterizing both inflammation and pain. We observed that inflammation and pain in flare 3 did not resolve by study termination (10 days post induction of flare 3) resulting in what appears to be a chronic phenotype. Interestingly, the cytokine expression profile in the flare 3 was similar to that observed in flare 2 with an up-regulation of IL-1β, IL-6, MCP-1 and CINC-1.

The efficacy of etanercept in the chronic phase of the model was also investigated. As observed in flare 2, prophylactic treatment in flare 3 with etanercept resulted in a significant inhibition of paw swelling, with a modest impact on pain which was statistically not significant. This data suggests that TNF may play similar role in flare 3. However, further investigation is required to delineate additional mechanisms that may be involved in the regulation of pain in this model.

Interestingly, the cohort of SCW treated rats that had received etanercept treatment in flare 2, when treated with the same dose of etanercept in flare 3, did not respond to the treatment. The lack of efficacy observed in flare 3 was attributed to the reduced systemic exposure of etanercept in these rats most likely due to the immunogenicity. Immunogenicity toward certain biologics, especially anti-TNF agents has been demonstrated in rodents as well as in the clinic [58, 59]. In addition, multiple dosing of the agent may also contribute to the generation of anti-drug antibodies [59]. Administration of anti-TNF alone in RA patients can elicit autoantibodies resulting in an enhanced clearance and loss of efficacy of the agent [60]. Species specificity may also contribute to the immunogenicity of biologic agents, as observed with certain chimeric antibodies [61, 62]. Interestingly, co-administration of methotrexate (MTX), along with certain anti-TNF agents, can reduce the immunogenicity and improve the efficacy of these agents in the clinic [63]. Although the mechanisms involved in the immunogenicity of biologic agents are not fully elucidated, anti-TNF in combination with MTX is considered to be a gold standard therapy for moderate to severe RA patients [64]. Hence, it will be interesting to test etanercept and MTX combination therapy compared to etanercept treatment alone in flare 3. The flaring mechanism in the SCW model allows for drug washout periods in between compound administration. This might provide useful preclinical insights on potential immunogenicity mechanisms that may be relevant in a clinical setting.

## Conclusions

In summary, we have described a novel extended mono-arthritic SCW multi-flare model with an additional flare that captures certain clinical aspects of RA. We report for the first time that this model can be used to simultaneously evaluate mechanical pain and inflammation. In addition, we have characterized our model to identify various cytokines and cell types that could be key drivers of disease in this model. Using etanercept we have demonstrated that TNF plays a key role in the onset and progression of paw swelling in the SCW model and can also contribute to the development of pain. Based on our studies, we report that this model provides a novel tool for drug discovery to assess anti-rheumatic agents targeting inflammation and pain.

## Authors’ original submitted files for images

Below are the links to the authors’ original submitted files for images. Authors’ original file for figure 1 Authors’ original file for figure 2 Authors’ original file for figure 3 Authors’ original file for figure 4 Authors’ original file for figure 5 Authors’ original file for figure 6 Authors’ original file for figure 7 Authors’ original file for figure 8 Authors’ original file for figure 9 Authors’ original file for figure 10 Authors’ original file for figure 11 Authors’ original file for figure 12 Authors’ original file for figure 13 Authors’ original file for figure 14 Authors’ original file for figure 15 Authors’ original file for figure 16 Authors’ original file for figure 17 Authors’ original file for figure 18 Authors’ original file for figure 19 Authors’ original file for figure 20
