# Supplementary figures and images for: Etanercept ameliorates inflammation and pain in a novel mono-arthritic multi-flare model of streptococcal cell wall induced arthritis
Source: BMC Musculoskelet Disord. 2014 Dec 4;15:409. doi: 10.1186/1471-2474-15-409 (PMC4320526; doi:10.1186/1471-2474-15-409)

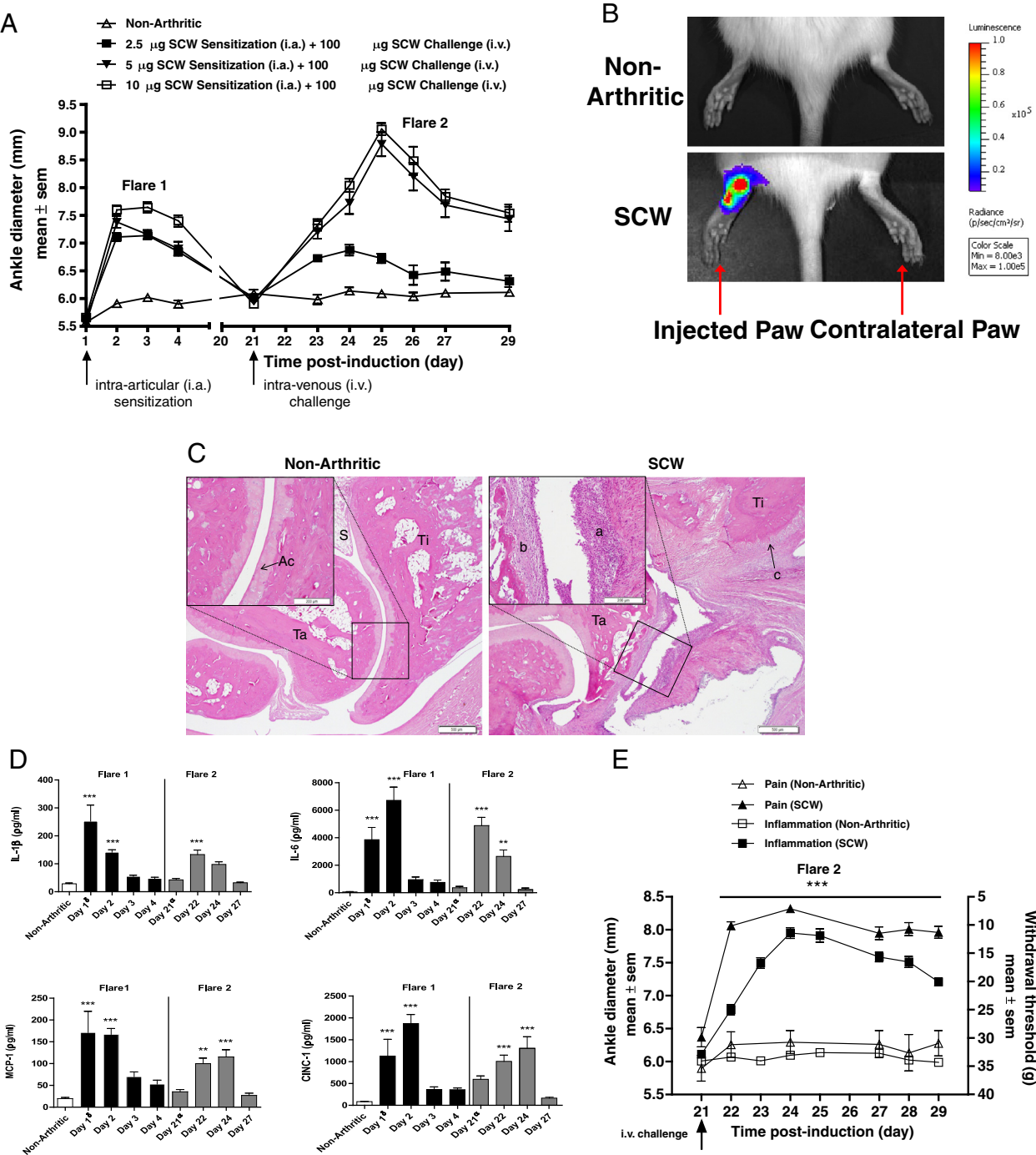

Supplement: Supplementary file 1 — Authors’ original file for figure 1 [file 12891_2014_2395_MOESM1_ESM.pdf]

A

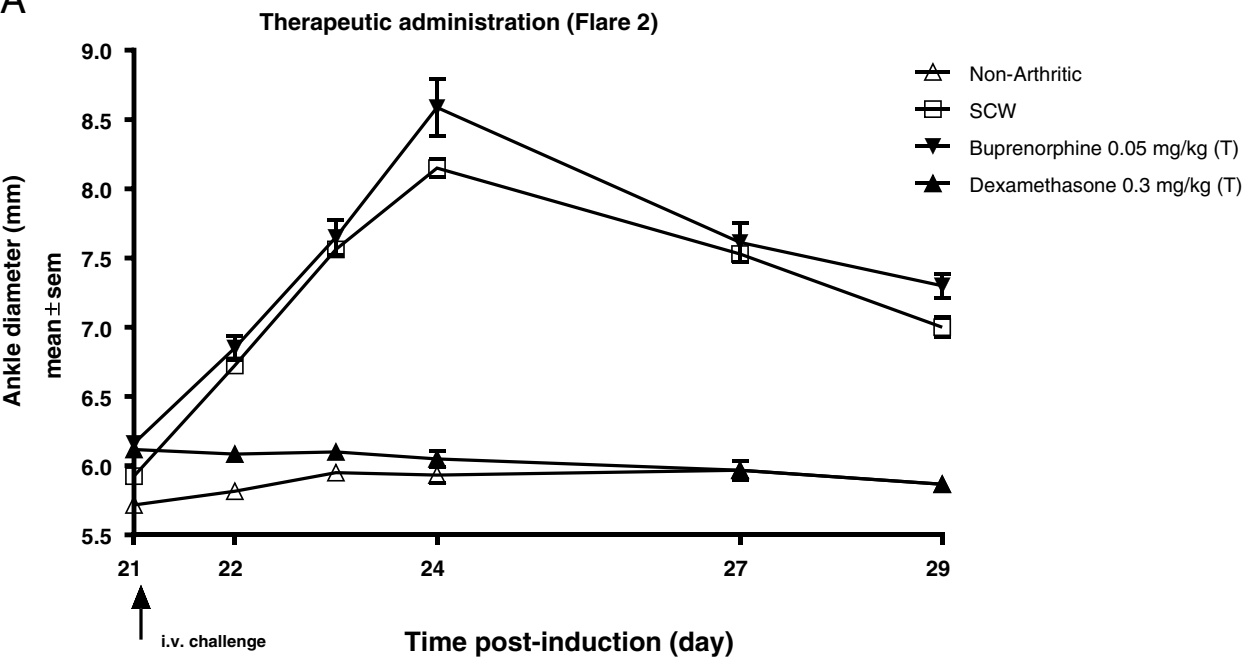

B

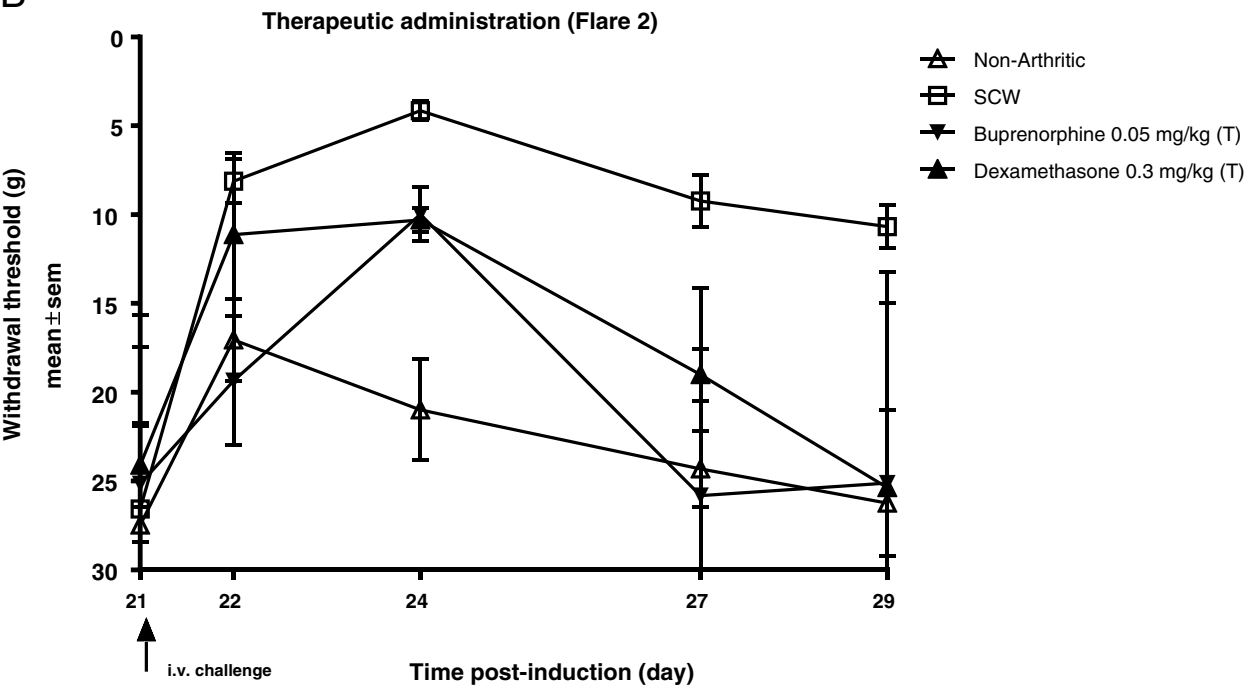

Supplement: Supplementary file 2 — Authors’ original file for figure 2 [file 12891_2014_2395_MOESM2_ESM.pdf]

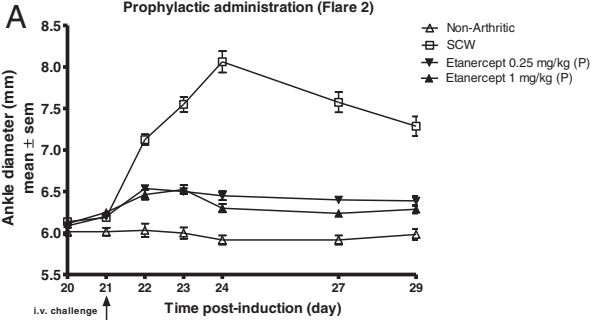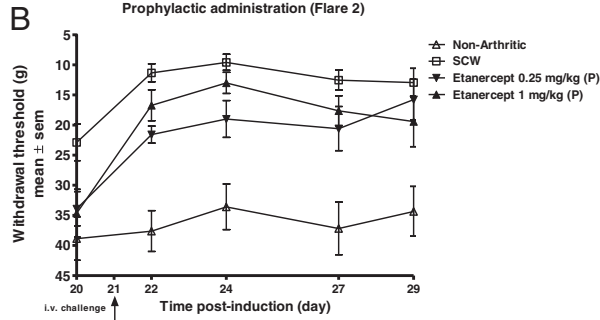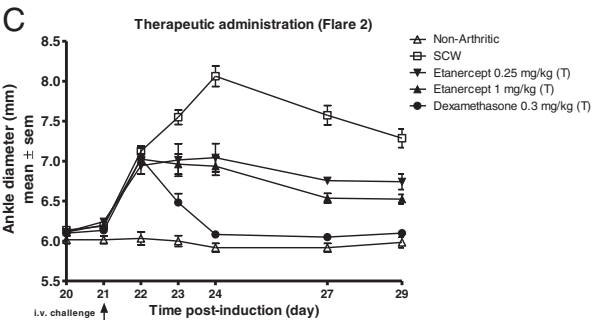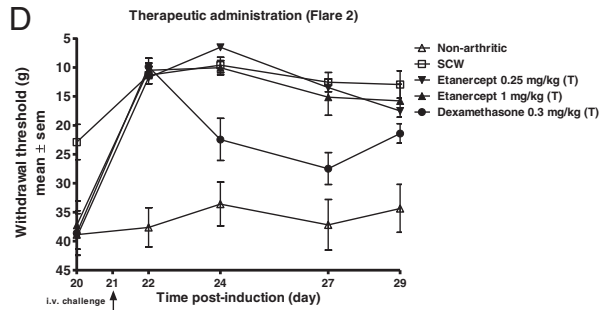

Supplement: Supplementary file 3 — Authors’ original file for figure 3 [file 12891_2014_2395_MOESM3_ESM.pdf]

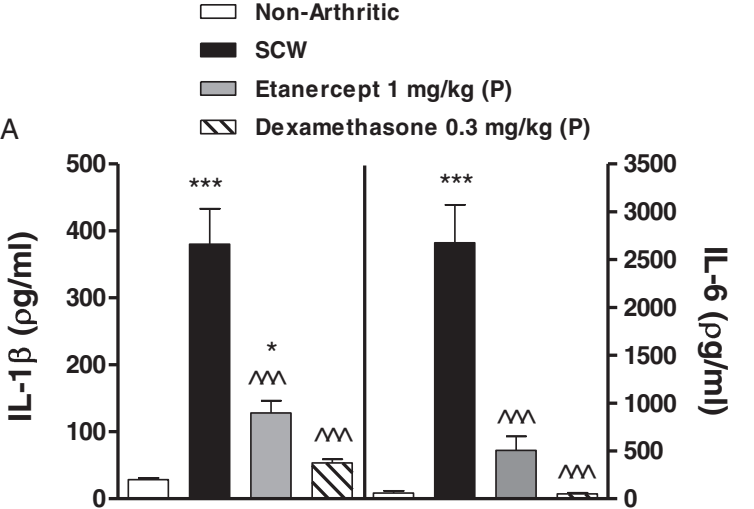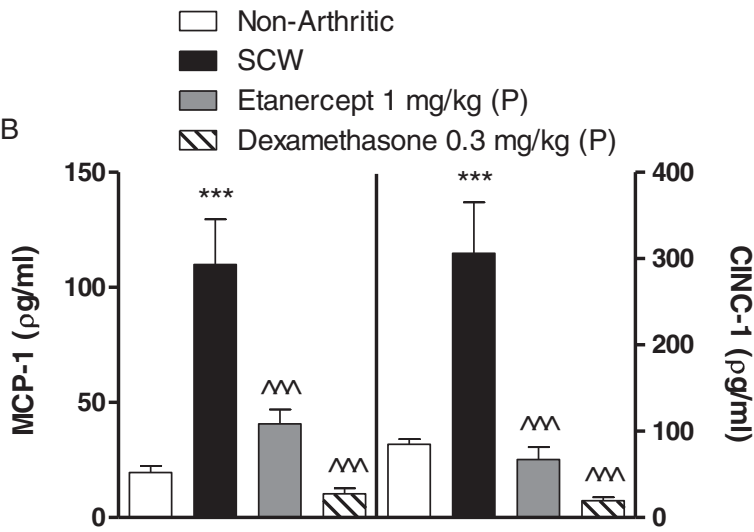

Supplement: Supplementary file 4 — Authors’ original file for figure 4 [file 12891_2014_2395_MOESM4_ESM.pdf]

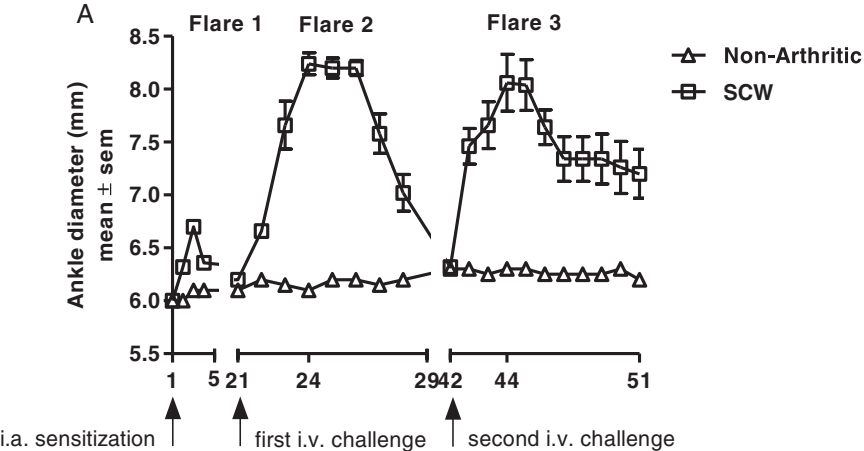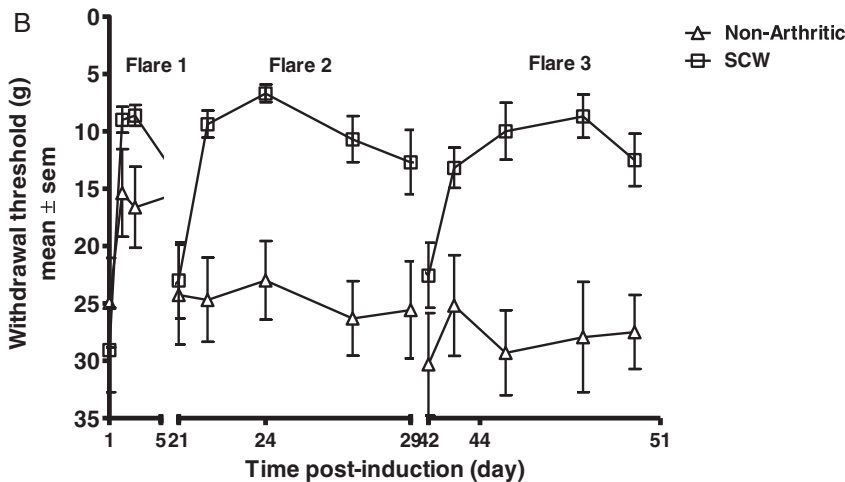

Supplement: Supplementary file 5 — Authors’ original file for figure 5 [file 12891_2014_2395_MOESM5_ESM.pdf]

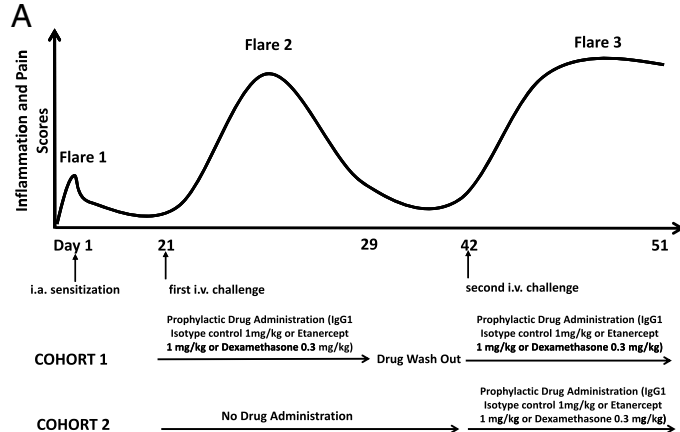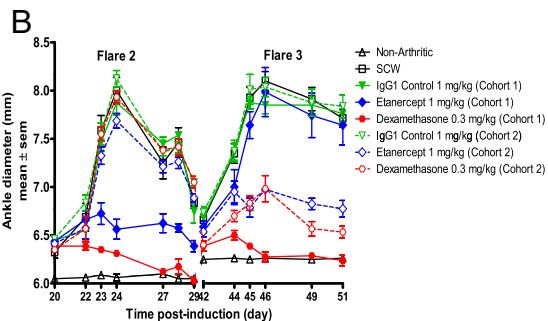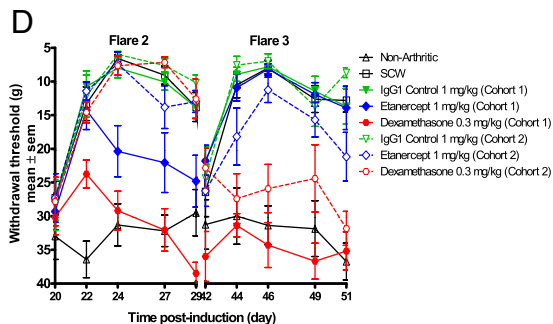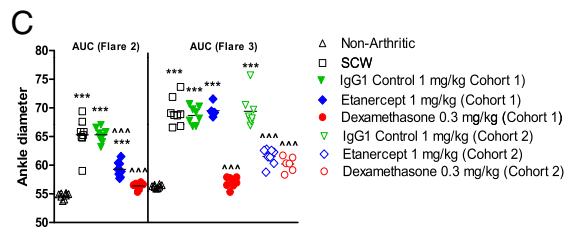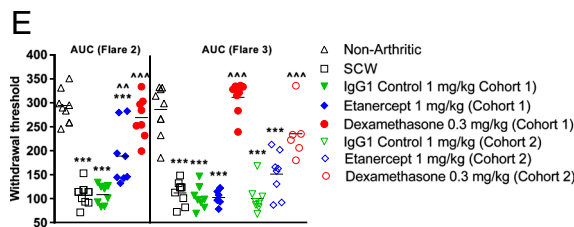

Supplement: Supplementary file 6 — Authors’ original file for figure 6 [file 12891_2014_2395_MOESM6_ESM.pdf]

## Slide 1
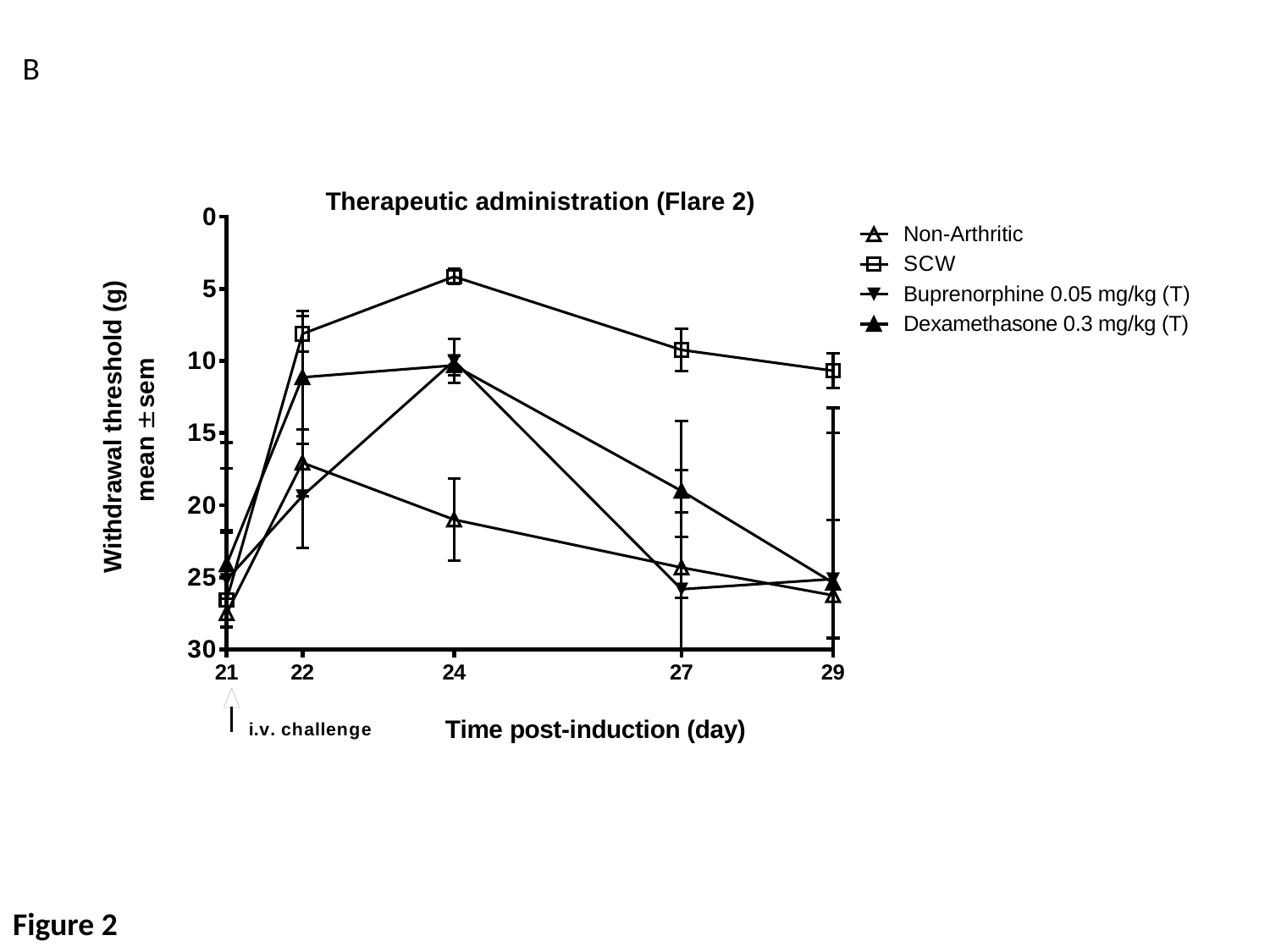

B
Figure 2

Supplement: Supplementary file 7 — Authors’ original file for figure 7 [file 12891_2014_2395_MOESM7_ESM.pptx]

## Slide 1
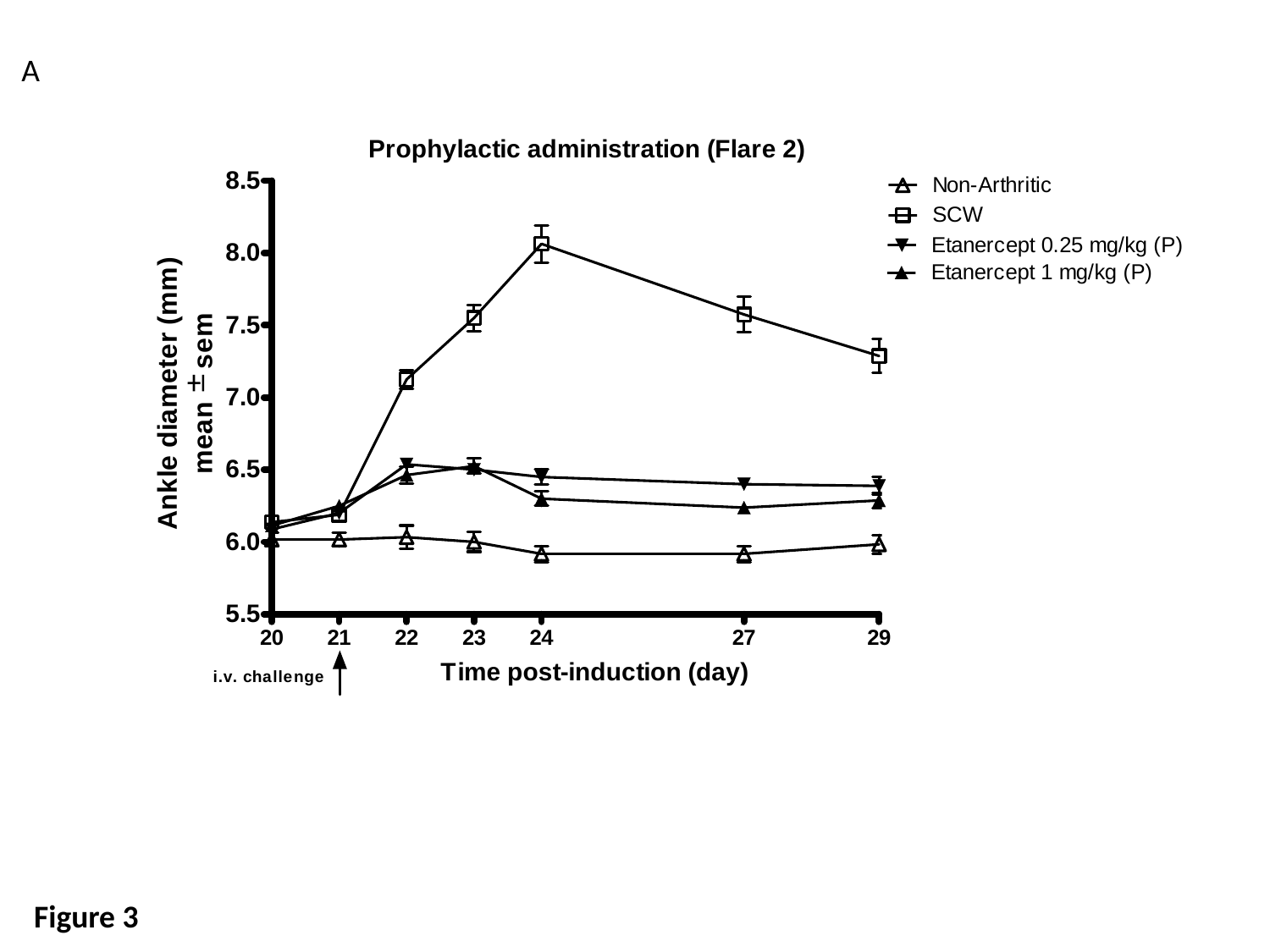

A
Figure 3

Supplement: Supplementary file 8 — Authors’ original file for figure 8 [file 12891_2014_2395_MOESM8_ESM.pptx]

## Slide 1
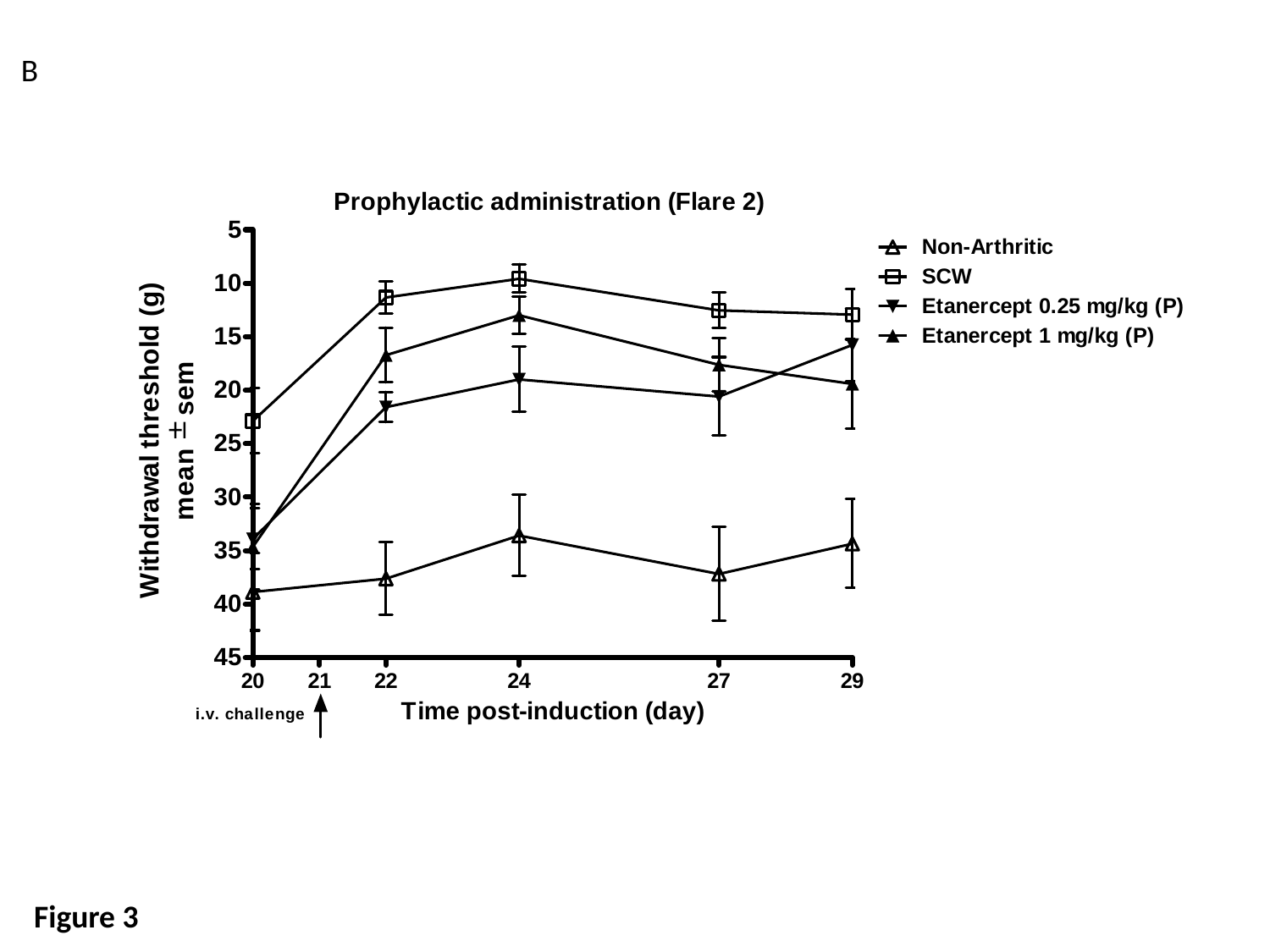

B
Figure 3

Supplement: Supplementary file 9 — Authors’ original file for figure 9 [file 12891_2014_2395_MOESM9_ESM.pptx]

## Slide 1
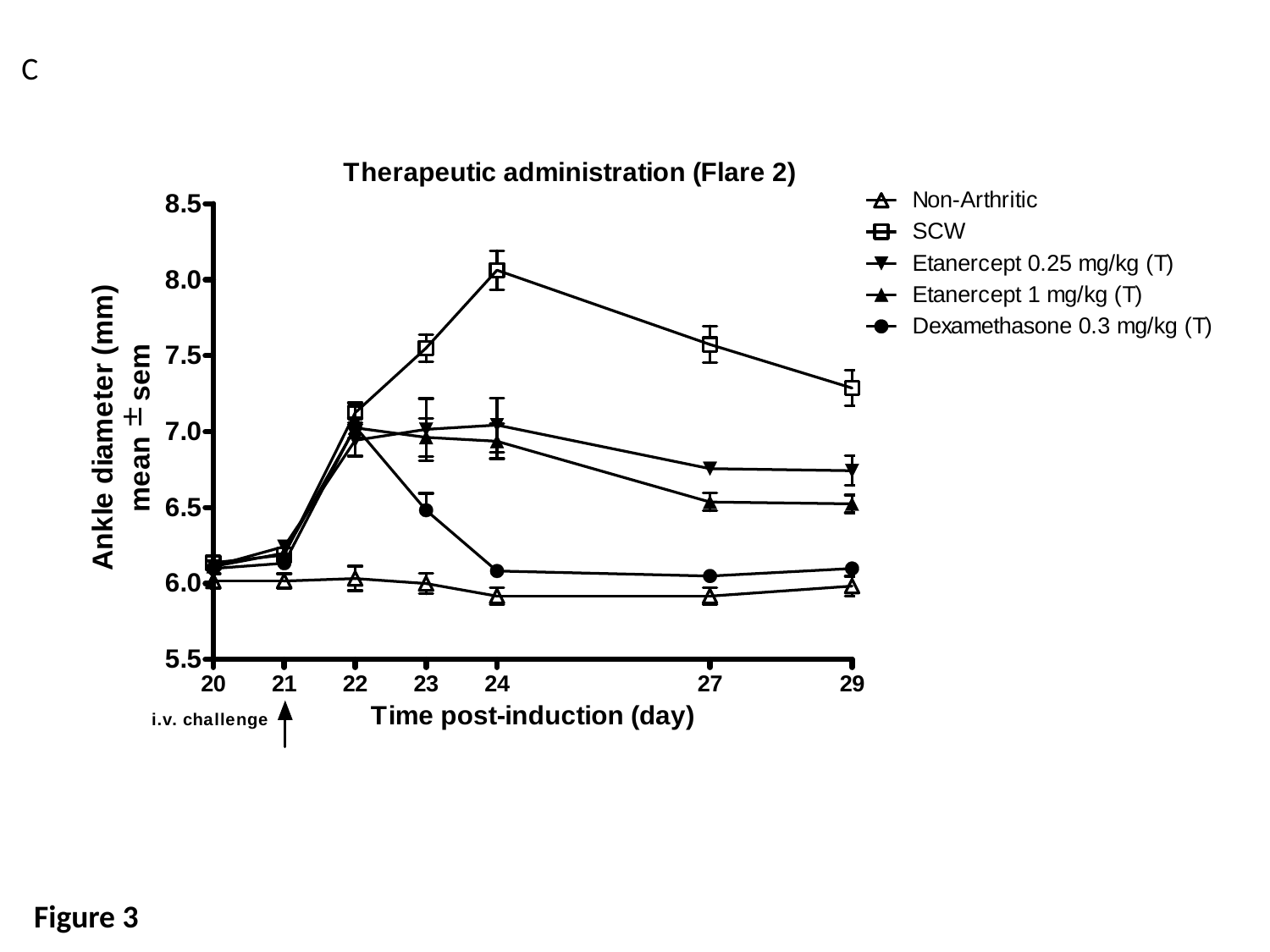

C
Figure 3

Supplement: Supplementary file 10 — Authors’ original file for figure 10 [file 12891_2014_2395_MOESM10_ESM.pptx]

## Slide 1
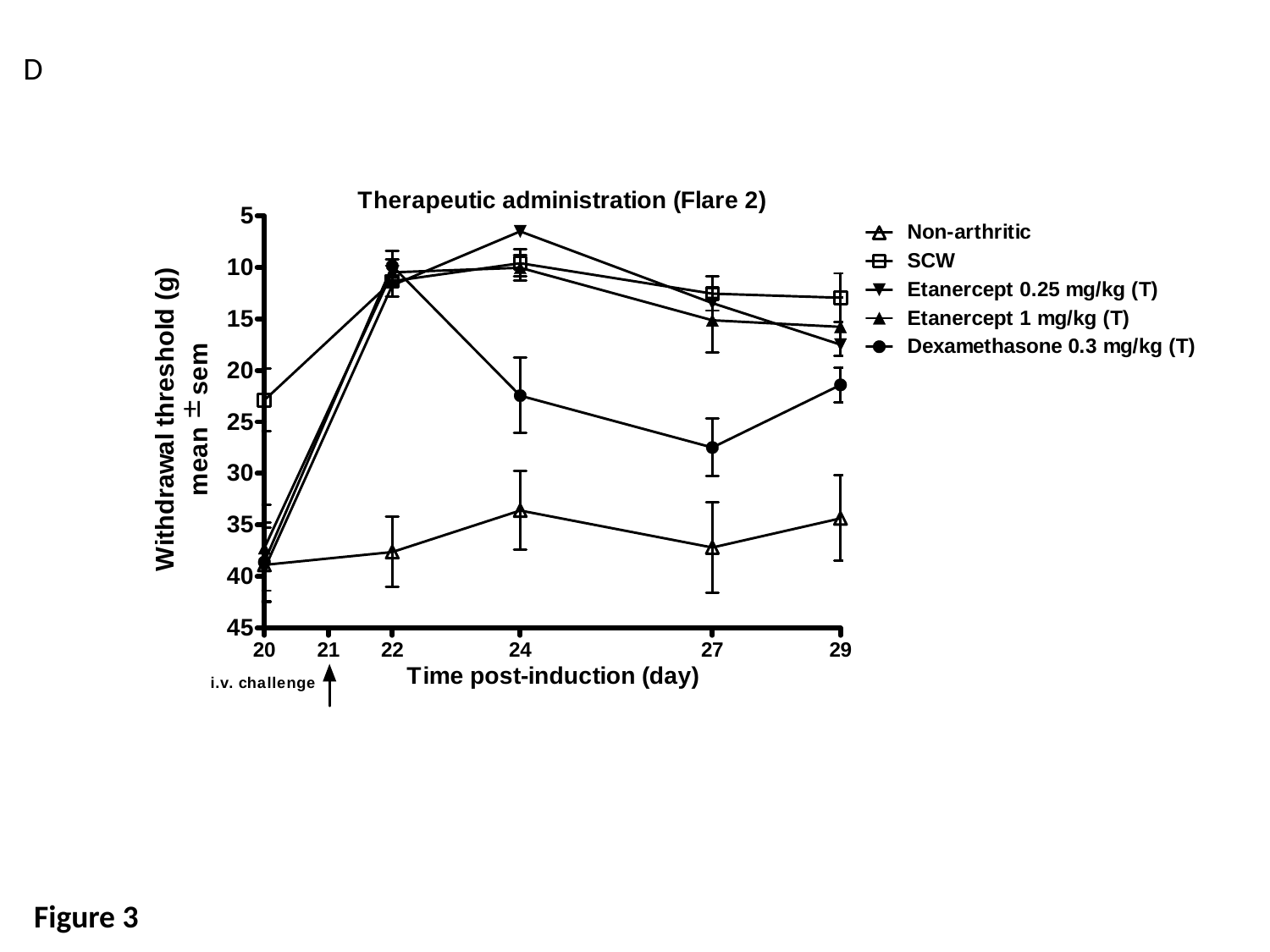

D
Figure 3

Supplement: Supplementary file 11 — Authors’ original file for figure 11 [file 12891_2014_2395_MOESM11_ESM.pptx]

## Slide 1
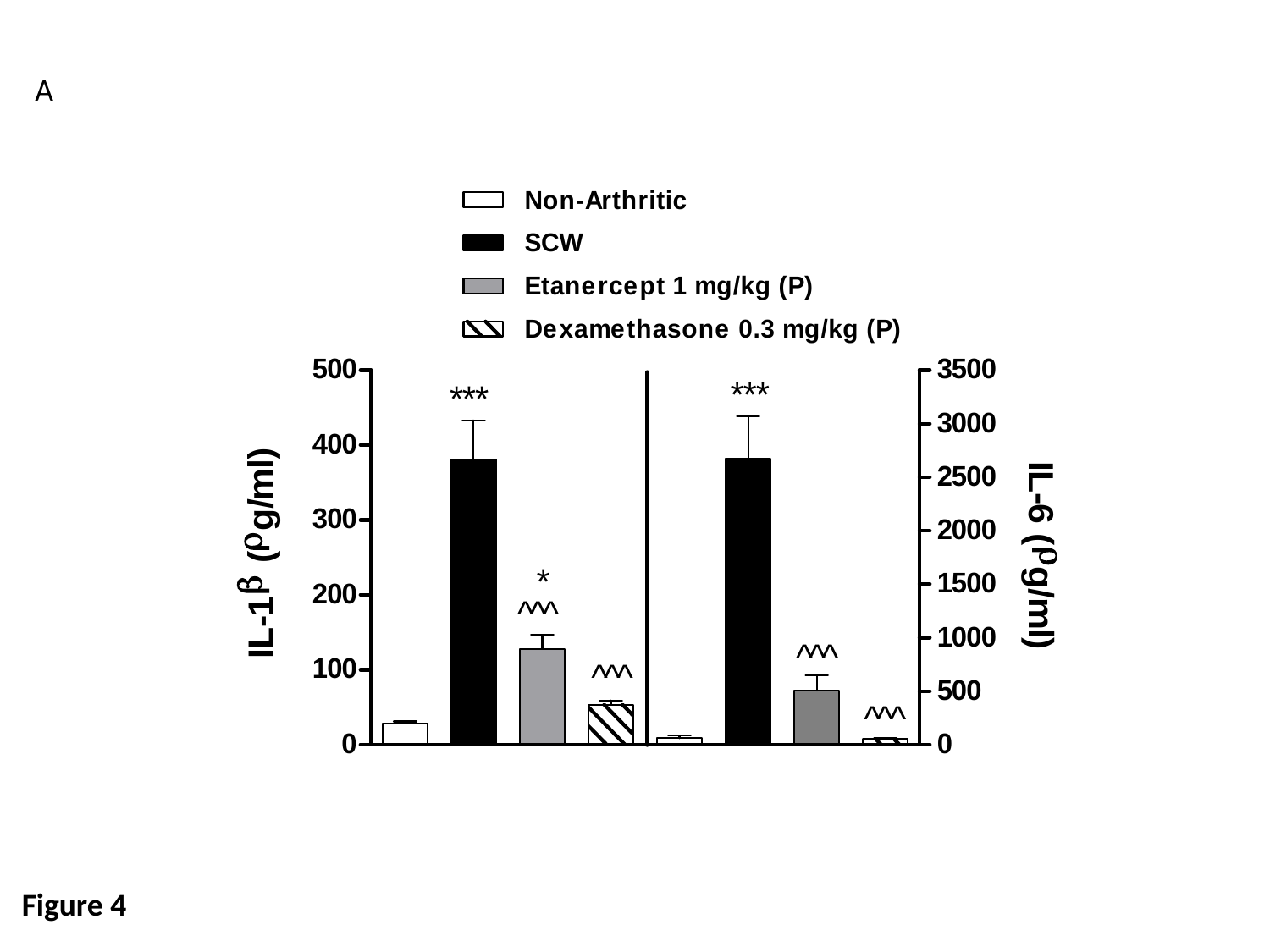

A
Figure 4

Supplement: Supplementary file 12 — Authors’ original file for figure 12 [file 12891_2014_2395_MOESM12_ESM.pptx]

## Slide 1
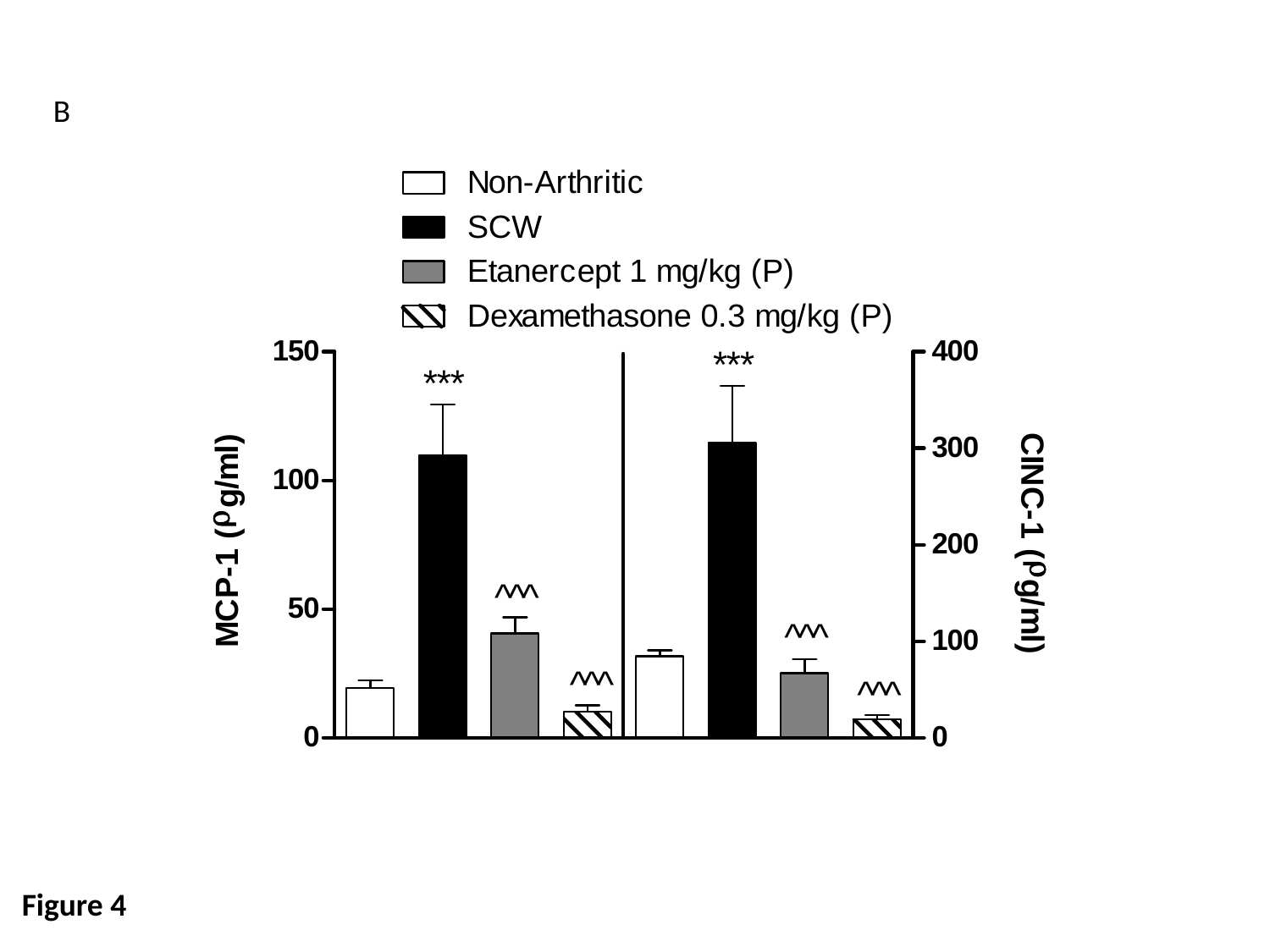

B
Figure 4

Supplement: Supplementary file 13 — Authors’ original file for figure 13 [file 12891_2014_2395_MOESM13_ESM.pptx]

## Slide 1
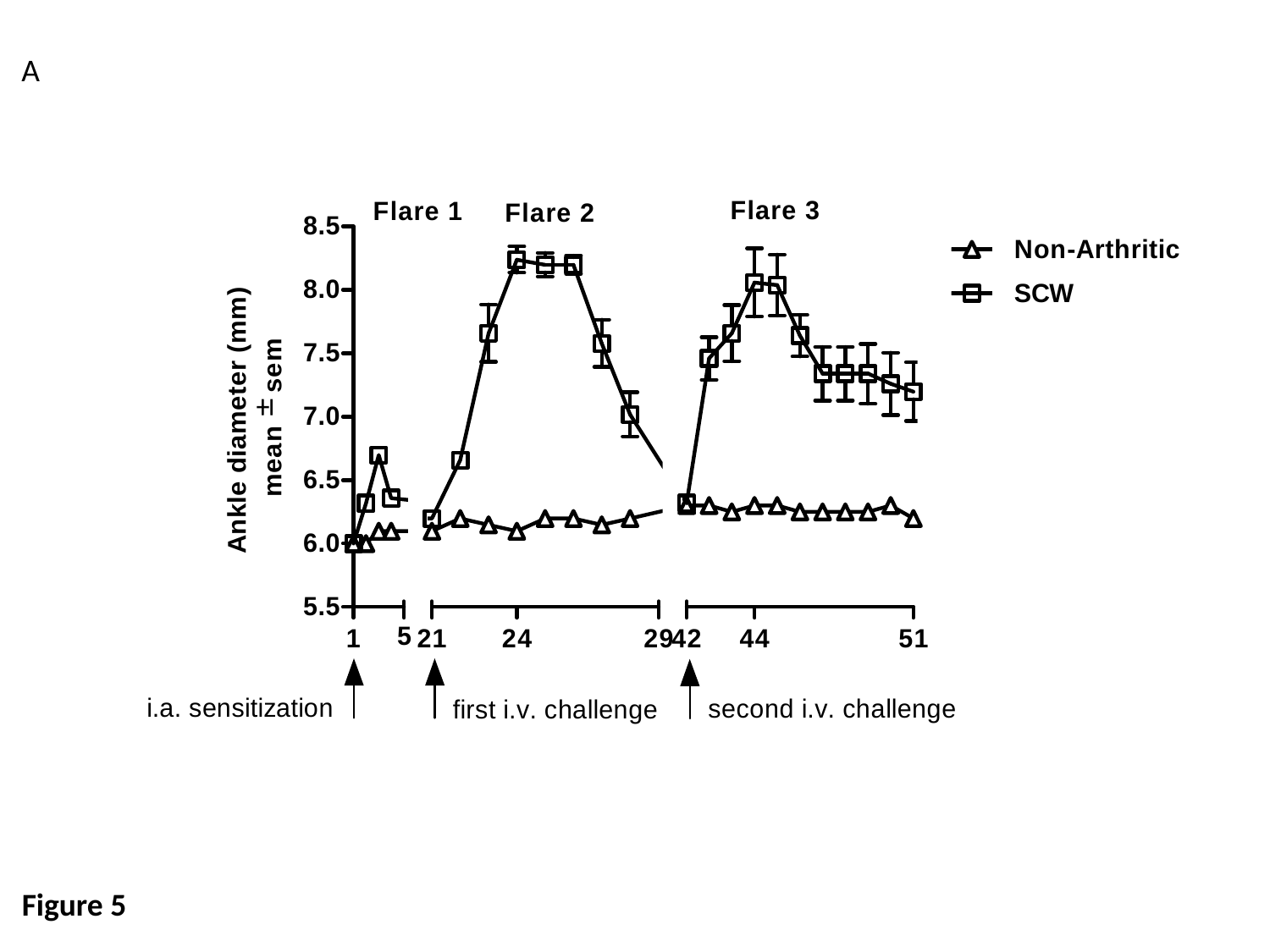

A
Figure 5

Supplement: Supplementary file 14 — Authors’ original file for figure 14 [file 12891_2014_2395_MOESM14_ESM.pptx]

## Slide 1
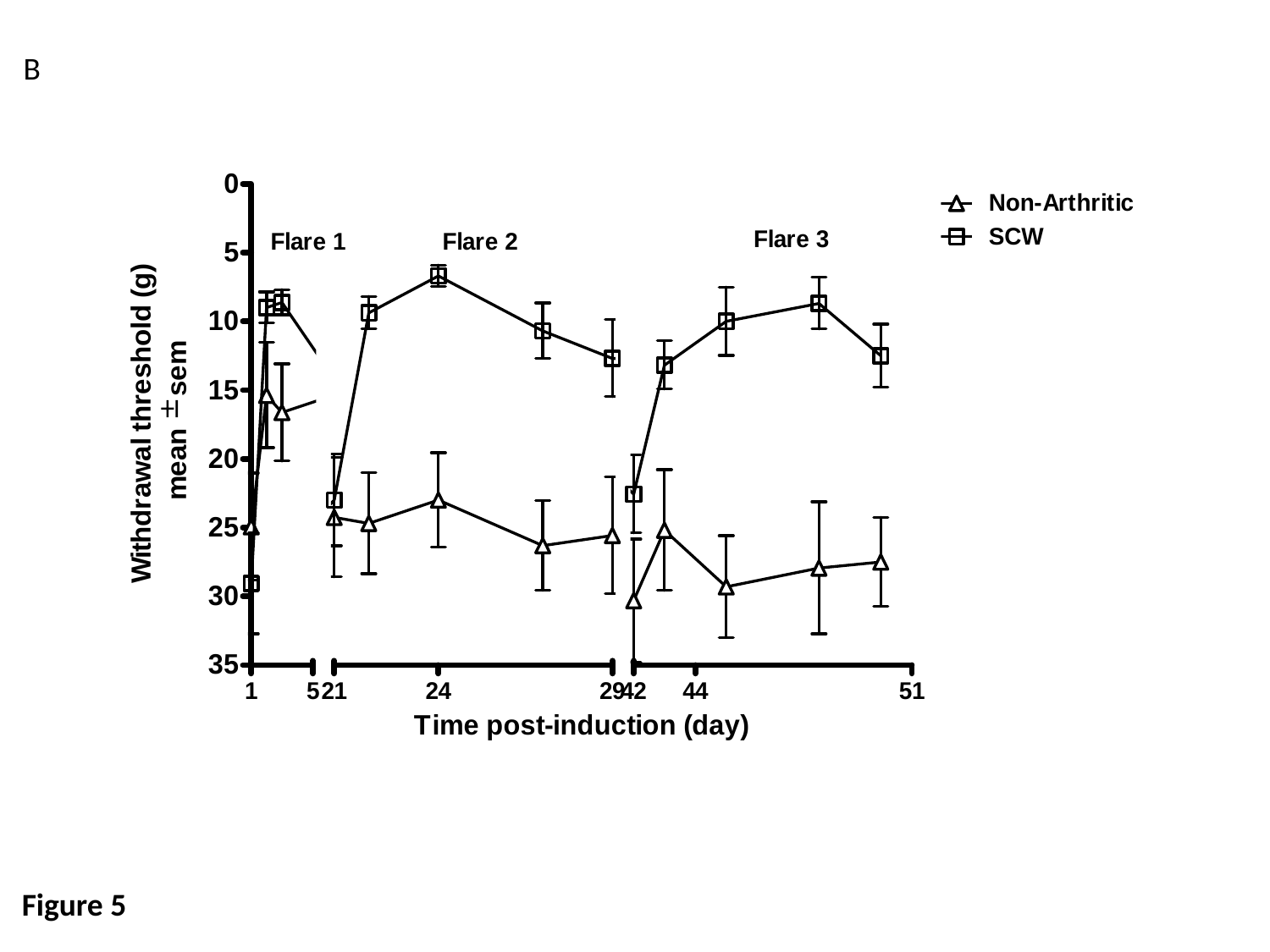

B
Figure 5

Supplement: Supplementary file 15 — Authors’ original file for figure 15 [file 12891_2014_2395_MOESM15_ESM.pptx]

## Slide 1
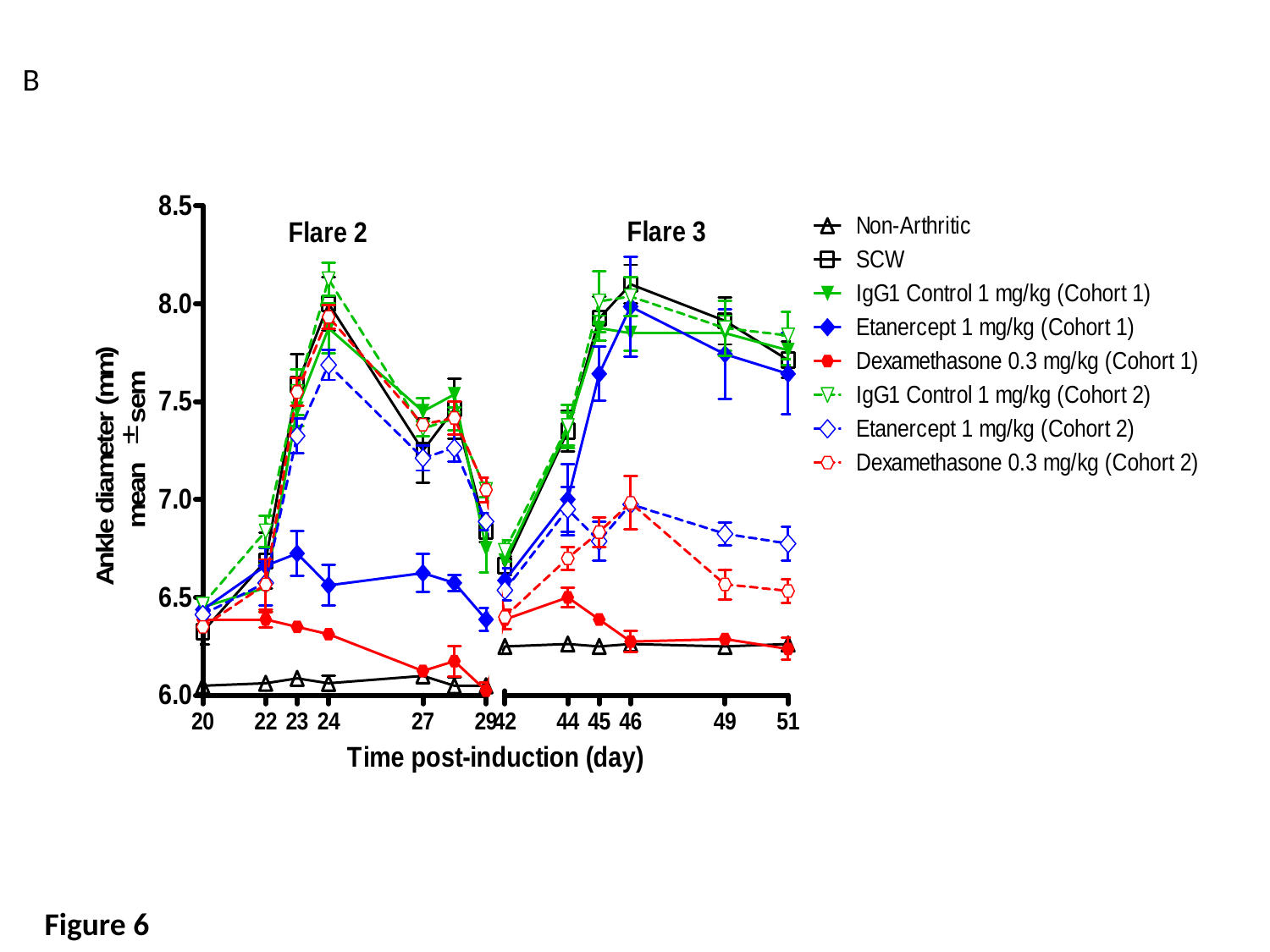

B
Figure 6

Supplement: Supplementary file 17 — Authors’ original file for figure 17 [file 12891_2014_2395_MOESM17_ESM.pptx]

## Slide 1
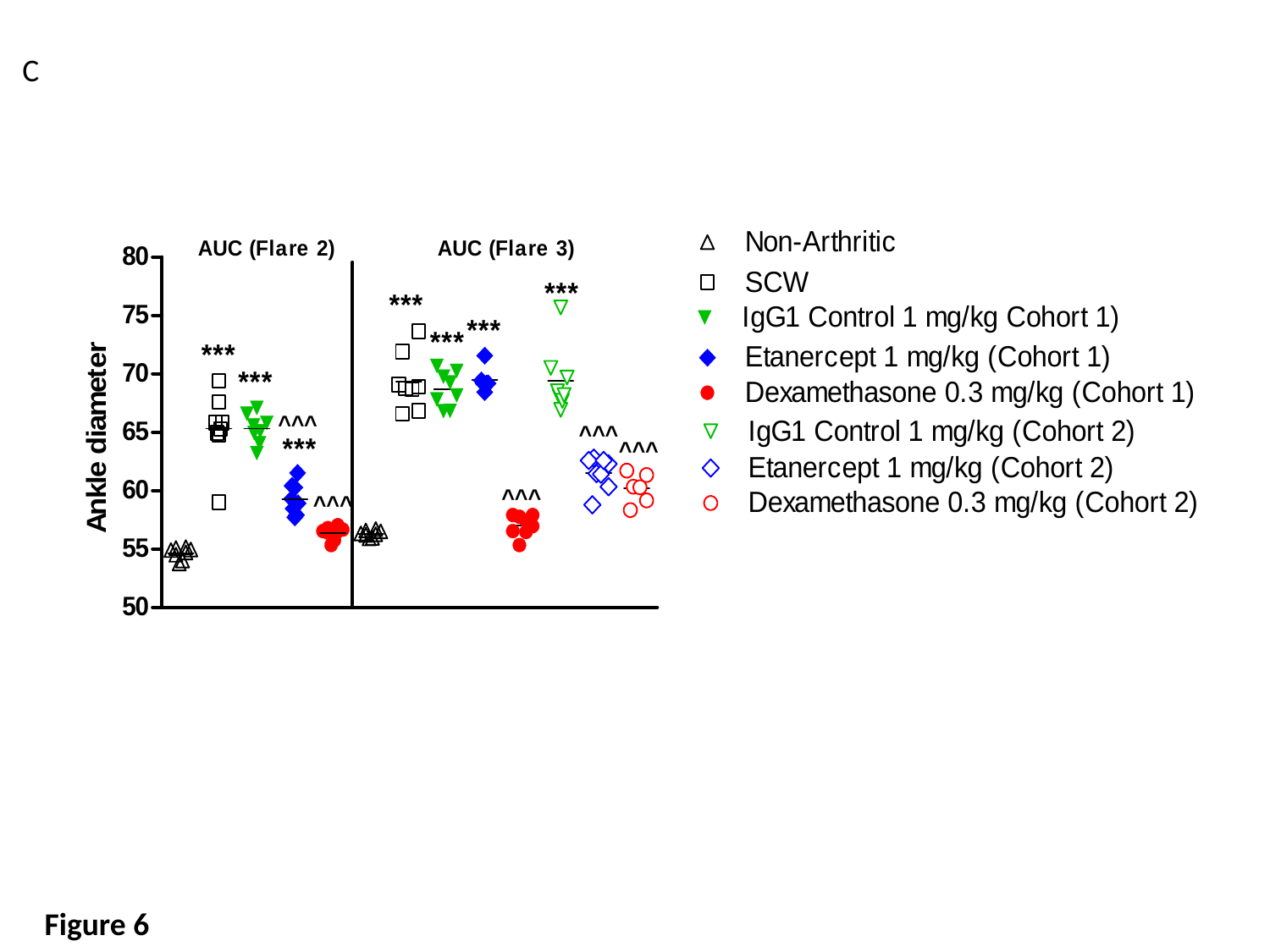

C
Figure 6

Supplement: Supplementary file 18 — Authors’ original file for figure 18 [file 12891_2014_2395_MOESM18_ESM.pptx]

## Slide 1
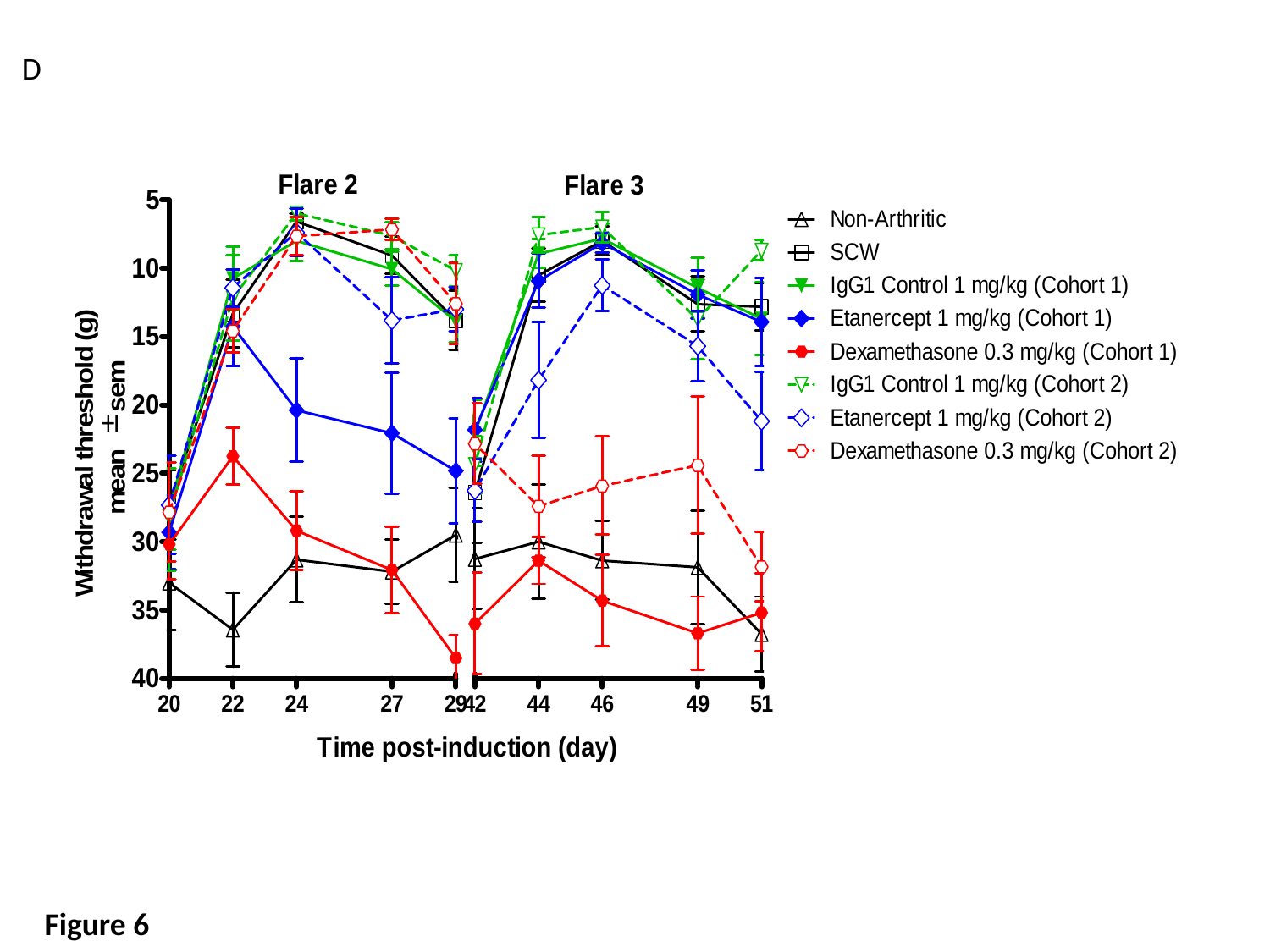

D
Figure 6

Supplement: Supplementary file 19 — Authors’ original file for figure 19 [file 12891_2014_2395_MOESM19_ESM.pptx]

## Slide 1
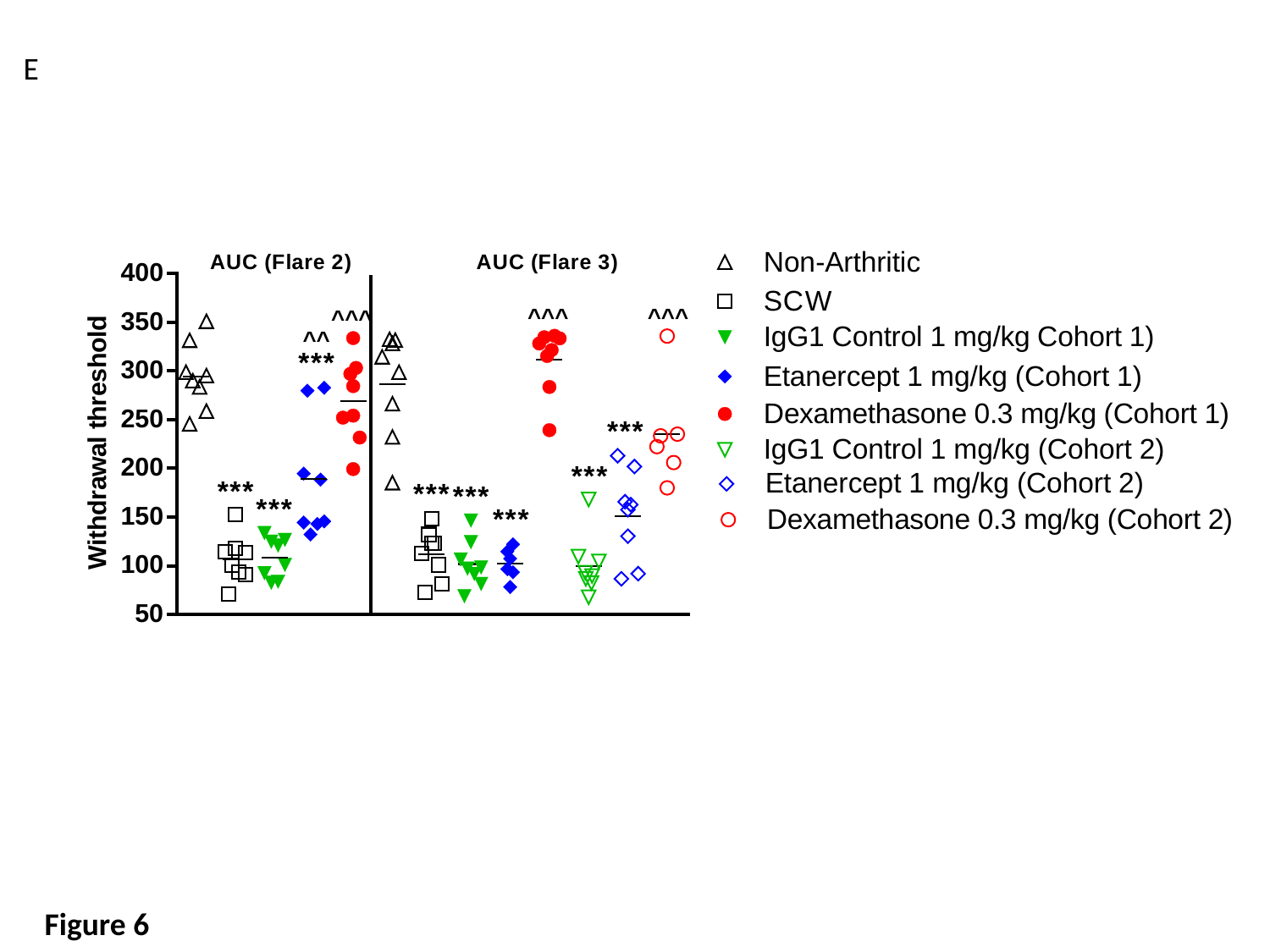

E
Figure 6

Supplement: Supplementary file 20 — Authors’ original file for figure 20 [file 12891_2014_2395_MOESM20_ESM.pptx]
