# Supplementary material for: Etanercept ameliorates inflammation and pain in a novel mono-arthritic multi-flare model of streptococcal cell wall induced arthritis
Source: BMC Musculoskelet Disord. 2014 Dec 4;15:409. doi: 10.1186/1471-2474-15-409 (PMC4320526; doi:10.1186/1471-2474-15-409)
Supplement: Supplementary file 16 — Authors’ original file for figure 16 [file 12891_2014_2395_MOESM16_ESM.pptx]

## Slide 1
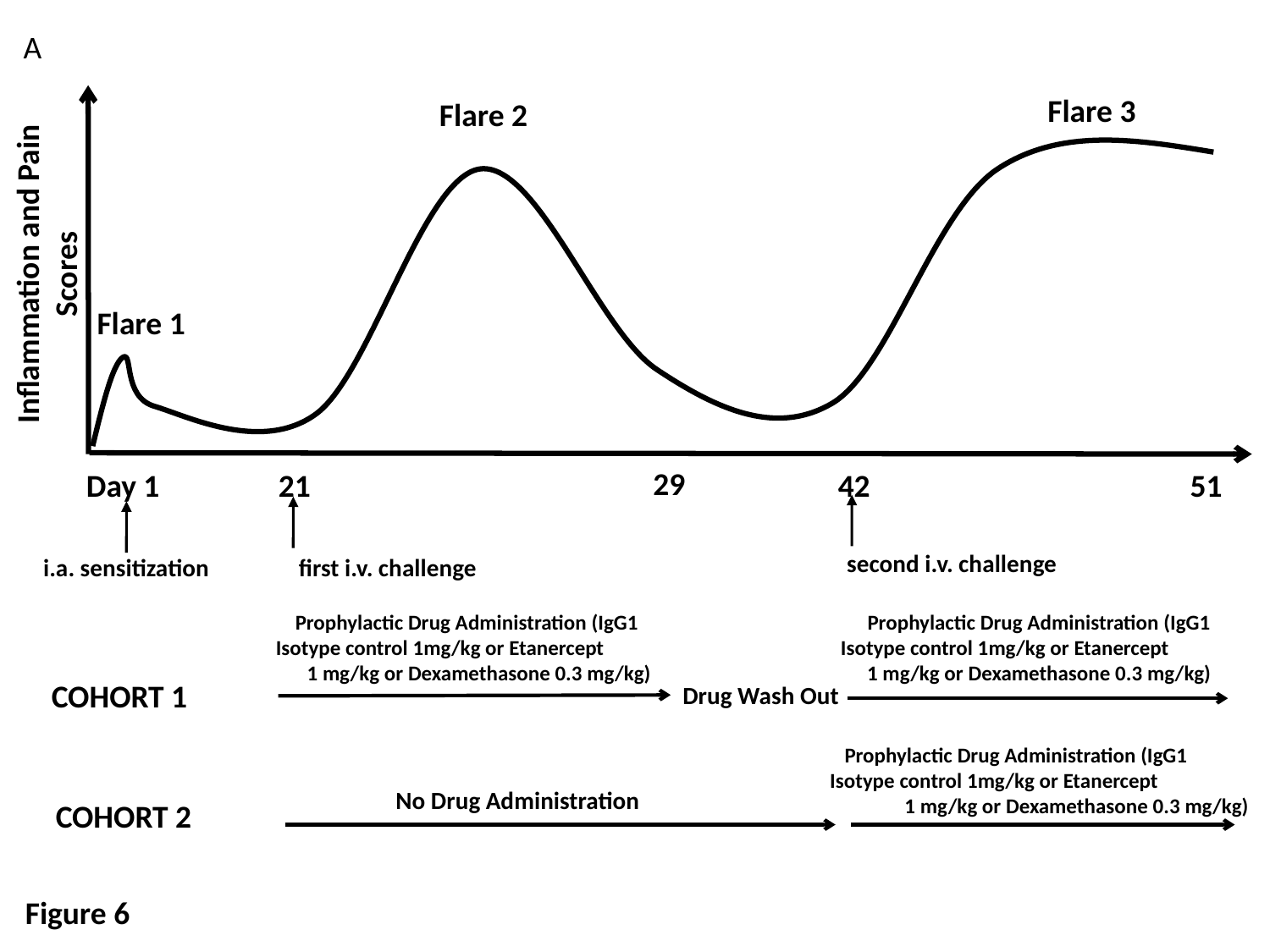

A
Flare 3
Flare 2
Inflammation and Pain Scores
Flare 1
29
Day 1
21
42
51
second i.v. challenge
i.a. sensitization
first i.v. challenge
Prophylactic Drug Administration (IgG1 Isotype control 1mg/kg or Etanercept 1 mg/kg or Dexamethasone 0.3 mg/kg)
Prophylactic Drug Administration (IgG1 Isotype control 1mg/kg or Etanercept 1 mg/kg or Dexamethasone 0.3 mg/kg)
COHORT 1
Drug Wash Out
Prophylactic Drug Administration (IgG1 Isotype control 1mg/kg or Etanercept 1 mg/kg or Dexamethasone 0.3 mg/kg)
No Drug Administration
COHORT 2
Figure 6
